# STAG-GuardNet: UAV-Assisted Spatio-Temporal Attack Graph Learning for Secure IoT Communication in Smart EV Charging Networks

**DOI:** 10.3390/s26185898

**Published:** 2026-09-17

**Authors:** Abdulrahman A. Alshdadi

**Affiliations:** Department of Information Systems and Technology, College of Computer Science and Engineering, University of Jeddah, Asfan Road, Jeddah 21959, Makkah, Saudi Arabia; alshdadi@uj.edu.sa

**Keywords:** UAV-assisted monitoring, DDoS detection, distributed denial-of-service attacks, smart electric vehicle charging networks, spatio-temporal graph learning, cyber-physical security, intrusion detection, metaheuristic optimization

## Abstract

Smart electric vehicle (EV) charging infrastructures are evolving into large-scale cyber-physical Internet of Things (IoT) systems that depend on distributed communication, real-time sensing, and spatially coordinated charging operations. However, their interconnected communication architecture exposes charging stations, EV communication links, and network gateways to coordinated distributed denial-of-service (DDoS) attacks. Existing intrusion detection approaches primarily rely on localized or static traffic analysis and therefore have limited capability to capture spatially distributed and temporally evolving attack behavior. This study proposes the Spatio-Temporal Attack Graph Guard Network (STAG-GuardNet), an unmanned aerial vehicle (UAV)-assisted spatio-temporal attack graph learning framework for DDoS detection and security monitoring in smart EV charging networks. The framework integrates spatiotemporal signal conditioning, telemetry-adaptive graph aggregation, temporal dependency learning, attack-memory encoding, and adaptive risk-aware attention to model coordinated cyber-physical attack behavior. UAV-assisted telemetry provides complementary spatial and wireless information on communication instability, signal variation, neighboring congestion, and distributed attack-related behavior. A Hybrid Hawk–Manta Adaptive Optimizer (HHMAO) is employed to improve hyperparameter selection and convergence stability under imbalanced, heterogeneous, and nonstationary traffic conditions. The framework is evaluated using a smart-city EV charging cybersecurity dataset and three benchmark IoT intrusion detection datasets, namely TON_IoT, Edge-IIoTset, and X-IIoTID. Experimental results show that STAG-GuardNet achieves 97.7% accuracy, a 97.7% weighted F1-score, and a 98.4% area under the receiver operating characteristic curve (AUC) on the primary dataset. The framework also maintains stable performance under noisy telemetry, missing observations, heterogeneous traffic distributions, and charging-node outages. These findings demonstrate the potential of STAG-GuardNet for resilient and spatially informed security monitoring in UAV-assisted IoT-enabled EV charging infrastructures.

## 1. Introduction

Electric-vehicle charging infrastructure in smart cities forms a cyber-physical environment in which charging stations integrate communication, authentication, billing, grid coordination, and real-time monitoring functions. This connectivity improves operational coordination but expands the cyberattack surface and exposes charging services and grid-facing communication interfaces to disruption [1]. Denial-of-service attacks can degrade charging availability, overload communication services, and interfere with coordinated vehicular operations; related studies have also examined resilient security control for autonomous vehicles operating under DoS attacks [2,3]. The growing integration of vehicle-to-grid (V2G), vehicular edge computing, and distributed communication further increases the importance of protecting dynamic vehicular communication and computation interfaces [4]. Recent studies have therefore emphasized cybersecurity risks associated with smart charging and V2G integration [5].

Data-driven intrusion detection has been widely investigated for protecting cyber-physical and IoT-enabled systems [6]. Existing EV-charging security studies use operational telemetry and communication behavior to identify anomalies and attack signatures [7]. However, many approaches rely primarily on localized or static traffic analysis, limiting their ability to represent coordinated attacks that evolve across multiple charging stations. Effective monitoring therefore requires joint modeling of temporal attack persistence and changing relationships among distributed charging nodes.

Recent studies have applied AI-based intrusion detection to EV charging security and spatiotemporal learning to distributed cyber-physical DDoS detection [8,9], while UAV-assisted intrusion detection and multi-UAV sensing–communication frameworks have been investigated for smart-city and IoT environments [10,11]. In the present dataset, UAV-assisted telemetry provides altitude, relative-distance, received signal strength indicator (RSSI) variation, signal-drift, and neighboring-congestion measurements that complement ground charging-station–EV (CS–EV) telemetry. Their integration requires heterogeneous-data fusion, spatial-context modeling, and temporal dependency learning. These characteristics motivate a security model that jointly represents changing communication relationships, distributed spatial context, and persistent attack behavior across EV charging infrastructure [12].

This work presents STAG-GuardNet as a UAV-assisted spatio-temporal learning framework for monitoring CS–EV communication behavior and detecting coordinated DDoS attacks across distributed smart-city charging infrastructures. The security objective is to identify attack-induced communication and service anomalies from the available cyber-physical telemetry while capturing their spatial coordination and temporal evolution. The proposed framework integrates spatiotemporal signal conditioning with telemetry-adaptive graph aggregation, temporal dependency learning, attack-memory encoding, and adaptive risk-aware attention to model coordinated attack behavior across distributed charging infrastructures. UAV-assisted telemetry provides complementary spatial and wireless context for communication instability and neighboring congestion. The Hybrid Hawk–Manta Adaptive Optimizer (HHMAO) is used for offline hyperparameter selection by combining HHO-based exploration with MRFO-based refinement. The framework has been designed to deliver resilient detection capabilities for DDoS attacks in EV charging systems that operate within intricate smart city infrastructures. STAG-GuardNet models coordinated DDoS behavior by jointly representing how communication relationships change across charging stations and how abnormal activity evolves over time. The charging-station-centered node set remains fixed, while directed edge membership and edge weights are recomputed from spatial affinity, communication similarity, and lagged abnormal-traffic behavior at each time step. The resulting graph representation is combined with temporal dependency learning and an attack-memory state that preserves persistent abnormal patterns across successive observations. UAV-assisted spatial and wireless measurements provide additional context for communication instability and neighboring congestion, while risk-aware attention emphasizes the most informative cyber-physical states during attack classification. The main contributions of this work are summarized as follows:

- A telemetry-dependent directed graph formulation is developed for distributed CS–EV monitoring, where spatial affinity, communication similarity, and lagged abnormal-traffic coupling jointly determine time-varying interaction relationships among charging-station-centered cyber-physical states.
- A spatio-temporal learning architecture combines telemetry-adaptive graph aggregation with temporal dependency learning and persistent attack-memory encoding, enabling the model to distinguish short-lived local disturbances from sustained and coordinated DDoS behavior.
- UAV-assisted spatial and wireless observations are integrated with ground CS–EV telemetry through risk-aware feature modulation, providing complementary information about communication instability, congestion overlap, and spatially distributed attack behavior.
- Comprehensive cross-dataset evaluation, ablation analysis, robustness assessment, and explainability studies demonstrate that the proposed framework consistently outperforms recent deep cyber-physical security models, demonstrating its effectiveness and computational practicality for intelligent DDoS detection in next-generation EV charging ecosystems.

The remainder of this paper is organized as follows. Section 2 reviews existing research on cyber-physical security and DDoS detection. Section 3 presents the proposed STAG-GuardNet framework, including the dataset, preprocessing, feature representation, model architecture, and optimization strategy. Section 4 reports the experimental setup, comparative results, ablation analysis, robustness assessment, explainability analysis, and computational-efficiency evaluation. Section 5 concludes the paper and outlines future research directions.

## 2. Related Work

Recent IoT security research also demonstrates that protection mechanisms extend beyond intrusion classification to communication confidentiality, where dynamic cryptographic schemes have been investigated for securing time-varying IoT data [13]. These approaches combine transformer learning, residual feature extraction, graph reasoning, and optimization strategies to represent complex attack patterns beyond conventional CNN–RNN pipelines. TransMalDE [14] serves as a transformer-based framework for hierarchical malware analysis to model multi-level behavioral patterns that improve IoT malware detection. The model shows effective long-range dependency capture capabilities, but it lacks spatial coordination functions between its distributed infrastructure nodes while concentrating on software-level artifacts. Transformer-based temporal modeling has also been applied to anomaly detection in safety-critical cyber-physical environments, as demonstrated by AF-TAD for multivariate aircraft fuel-system monitoring [15]. The STOA-DGAN [16] combines Zebra Optimization Algorithm-based feature selection with a dual-channel graph attention network and Sooty Tern Optimization Algorithm-based hyperparameter tuning for IoT botnet detection. STOA-DGAN employs stochastic optimization to boost DDoS and botnet detection performance, which increases system resistance against emerging threats [16]. The approach requires centralized training to succeed because it does not support distributed methods or infrastructure-aware deployment. Transformer-based hybrid architectures have also been investigated for IoT security. BEFNet [17] uses Bidirectional Encoder Representations from Transformers (BERT)-based feed-forward representation learning for IoT malware analysis, while its optimized BEFSONet variant applies metaheuristic hyperparameter optimization. The models maintain high semantic representation capabilities but function as sequence-level malware analysis tools that do not depict cyber-physical interactions. DLBITM-AMD [18] combines binary grey wolf feature selection with improved Transformer–RNN learning for Android malware detection in Internet-of-Vehicles environments. The model reduces feature redundancy and improves endpoint-level malware detection, but it does not explicitly represent distributed spatial relationships among cyber-physical infrastructure nodes.

The hybrid architecture ResDNViT uses its residual dense feature extraction system to create a vision-transformer-style attention mechanism that detects network intrusions based on network-flow information [19]. The model combines local feature refinement with global contextual learning, but its analysis remains based on network-flow representations. GraphFedAI combines graph neural networks with federated learning to support privacy-preserving, structure-aware intrusion detection without centralizing local training data [20]. The framework provides privacy protection, but it requires a stable graph topology and it does not explicitly model time-varying spatial interactions among distributed infrastructure nodes. Advanced transformer hybrids help identify industrial botnets. AdvBiTrans uses its bidirectional transformer modeling to create adversarial training methods that support feature clustering to boost evasion resistance in DDoS attacks [21]. The framework provides support for industrial IoT applications, yet it lacks the capacity to manage extensive urban infrastructure systems. Adaptive latent-space clustering and few-shot wavelet learning have also extended malware representation learning. The ALDC-FSWL methodology in [22] improves Android malware detection through latent-space dip clustering and wavelet-based few-shot representation learning, but it does not model spatiotemporal DDoS propagation across cyber-physical charging infrastructures. In [23], a hybrid deep detection framework with recursive feature reduction targets DDoS detection but is less adaptable to coordinated, time-varying assaults. IMDP-HDL [24] uses deep representation learning together with heuristic search methods to improve malware detection performance, but its evaluation process only applies to static datasets that undergo offline assessment.

Recent cyber-physical security studies have extended deep learning to networked energy and transportation infrastructures. HC-GRNN combines convolutional feature extraction with gated recurrent learning for DDoS traffic classification in software-defined networks [25], while DL-CADF applies deep learning to cyberattack detection in DC shipboard microgrids [26]. Federated LSTM–autoencoder learning has also been investigated for privacy-preserving anomaly detection in smart electric grids [27]. These approaches demonstrate the effectiveness of temporal and distributed learning for cyber-physical security; however, they do not jointly integrate UAV-assisted telemetry, time-varying communication relationships, and coordinated DDoS behavior across distributed EV charging infrastructure.

Table 1 summarizes the principal contributions and limitations of the reviewed methods. Among them, the bidirectional recurrent attentive graph neural network (BiRAGNN) [28] provides the closest graph–temporal reference because it combines graph-based interaction modeling with recurrent learning for vehicle-to-grid communication analysis. BiRAGNN jointly represents spatial and sequential dependencies through a predefined probabilistic charging graph, whereas communication relationships in distributed charging environments may vary with wireless conditions, congestion, node behavior, and abnormal traffic activity. This distinction motivates telemetry-adaptive graph construction in which the retained relationships are recomputed from the observations available at each time step. Cross-layer intrusion detection has also been investigated for drone communication networks [29], further demonstrating the value of combining information from multiple communication and sensing layers.

Graph-based learning has also shown strong capability for representing interactions among distributed and time-varying physical entities. ParallelGraphNet uses graph-based multi-sensor representation and sensor optimization for fault diagnosis in complex physical infrastructure [30]. DADiffNet introduces delay-aware diffusion learning with adaptive subgraphs to represent time-varying inter-node dependencies in large-scale traffic networks [31], while hypergraph learning has been used to characterize higher-order interactions among intelligent vehicles and their surrounding traffic scenarios [32]. These studies demonstrate the value of adaptive graph structures for heterogeneous spatiotemporal data, although their objectives differ from coordinated DDoS detection in UAV-assisted EV charging infrastructure.

STAG-GuardNet addresses these requirements through a directed telemetry-adaptive graph in which spatial affinity, communication similarity, and lagged abnormal-traffic behavior determine the time-varying relationships among charging-station-centered cyber-physical states. Temporal dependency learning captures sequential behavior, while attack-memory encoding preserves persistent abnormal patterns across successive observations. UAV-assisted measurements contribute complementary spatial and wireless context to graph construction and risk assessment. The resulting representation therefore combines changing communication relationships, temporal persistence, and UAV-assisted cyber-physical information within a unified DDoS detection model.

## 3. Proposed System Model

**Figure 1:**
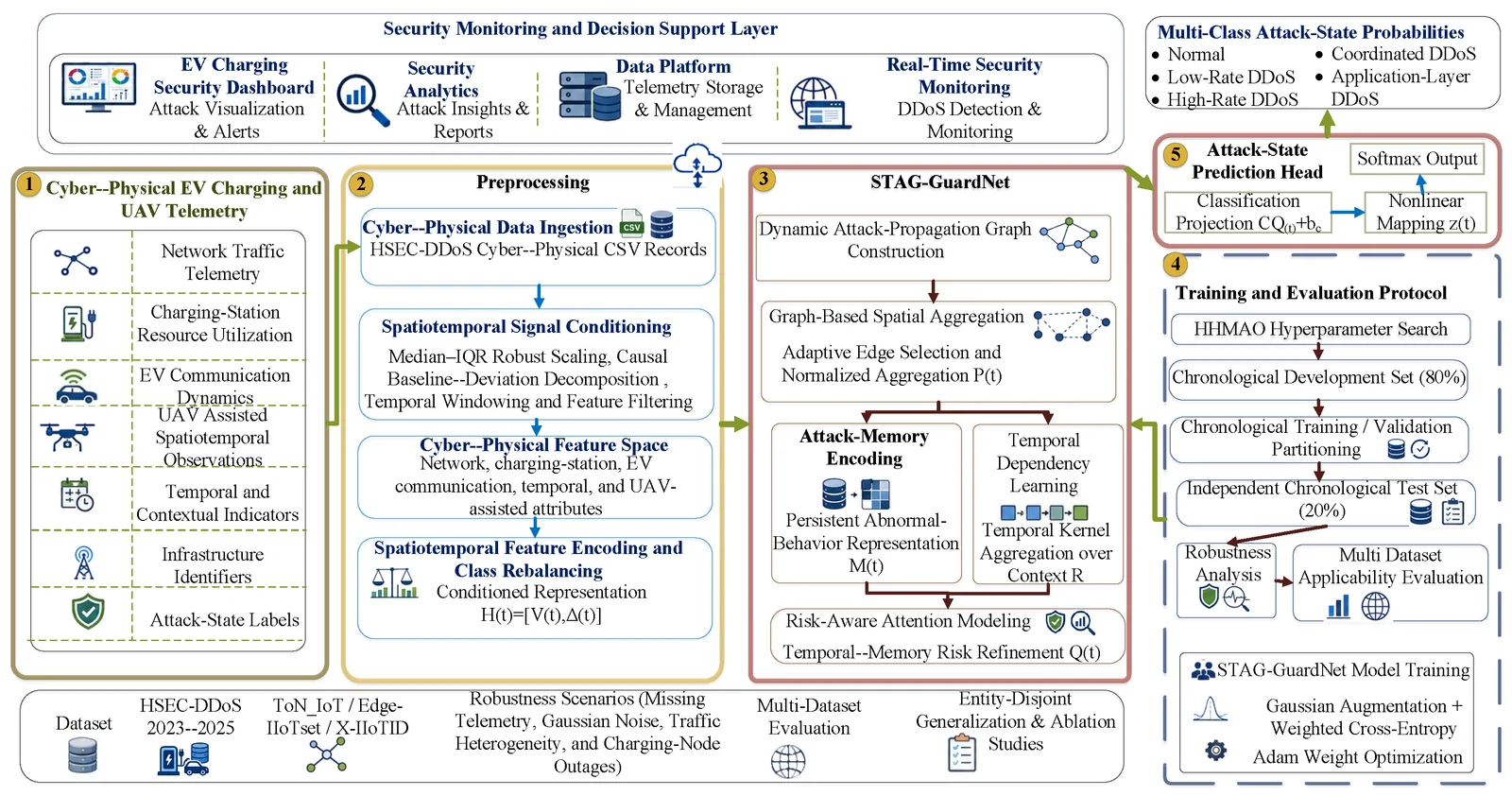
Framework of the proposed STAG-GuardNet for DDoS attack detection in smart city EV charging infrastructures.

This section describes the proposed Spatio-Temporal Attack Graph Guard Network (STAG-GuardNet) for DDoS attack detection in smart city EV charging infrastructures. The framework models the complex cyber-physical interactions among charging stations, EV communication systems, UAV-assisted monitoring units, and spatially distributed infrastructure nodes operating under dynamic urban traffic conditions. The proposed framework performs spatiotemporal traffic analysis through adaptive signal conditioning, telemetry-adaptive graph construction, temporal dependency learning, and risk-aware attention modeling within a unified learning architecture. Unlike conventional station-centric security models, the proposed method captures both localized anomalies and coordinated attack propagation patterns across distributed charging environments. The framework further integrates adaptive graph aggregation, attack-memory encoding, and hybrid metaheuristic optimization to improve detection robustness under heterogeneous and non-stationary traffic conditions. The complete DDoS detection pipeline operates from data preprocessing and feature representation to spatiotemporal attack graph learning and adaptive parameter optimization, as illustrated in Figure 1.

### 3.1. Threat Model and Security Assumptions

The security setting considered in STAG-GuardNet focuses on availability-oriented DDoS attacks against distributed EV charging communication and service infrastructure. The adversary aims to degrade or interrupt charging-related communication and service availability by generating abnormal traffic patterns that appear as Low-Rate DDoS, High-Rate DDoS, Coordinated DDoS, or Application-Layer DDoS activity. Attack traffic may originate from one or multiple malicious or compromised endpoints and may vary in intensity and coordination over time. The observable attack surface includes CS–EV communication links, charging-station processing resources, service interfaces, authentication queues, charging-authorization operations, and neighboring communication activity.

The defender observes the cyber-physical telemetry available to the monitoring pipeline, including packet-arrival behavior, SYN/ACK and UDP-flood activity, connection failures, charging-station CPU and memory utilization, API latency, authentication-queue length, EV handshake and retransmission behavior, charging-authorization delay, and UAV-assisted measurements. The UAV domain provides altitude, UAV-to-CS distance, RSSI fluctuation, signal drift, and neighboring-congestion overlap, allowing the detector to relate local communication anomalies to broader spatial and wireless conditions. The trained STAG-GuardNet model, model parameters, and monitoring pipeline are treated as trusted defender-side components, whereas the identities of the endpoints generating attack traffic are not required for classification.

The released telemetry does not encode the authentication state of individual communication channels or the identities of the original attack-generating hosts. Consequently, the detector operates on the communication and infrastructure observations available at the monitoring point without distinguishing authenticated and unauthenticated transport sessions. UAV telemetry is treated as an observed monitoring source in the evaluated setting. Missing measurements, Gaussian corruption, and charging-node outages are examined in the robustness analysis, whereas deliberate UAV telemetry spoofing, radio-frequency jamming, physical UAV takeover, training-data poisoning, model-parameter manipulation, credential compromise, and cryptographic attacks are not represented in the evaluated dataset. The resulting threat model therefore characterizes telemetry-based detection of DDoS activity and coordinated service disruption across the monitored EV charging infrastructure.

### 3.2. Dataset Description

STAG-GuardNet is evaluated using the publicly available Smart City EV Charging Network Spatiotemporal ost hoc Dataset [33], released through Zenodo as Version 1. The dataset contains 109,872 chronologically ordered observations recorded from 3 March 2023 to 3 April 2025 at 10 min intervals. The released data represent 60 charging-station identifiers, 191 distinct electric vehicle (EV) identifiers, and 8 unmanned aerial vehicle (UAV) identifiers, together with network, charging-infrastructure, EV-communication, temporal, and UAV-assisted telemetry.

Each record represents an aligned cyber-physical observation associated with a charging station, an EV, and a UAV monitoring entity. The recorded variables include packet-arrival behavior, packet-size statistics, SYN/ACK ratio, User Datagram Protocol (UDP) flood intensity, connection-request entropy, session-initiation failures, charging-station CPU and memory utilization, application programming interface (API) latency, authentication-queue length, EV handshake and retransmission behavior, charging-authorization delay, temporal indicators, and UAV-assisted measurements. The UAV-related variables comprise altitude, UAV-to-CS distance, received signal strength indicator (RSSI) fluctuation, signal-drift index, and neighboring-CS congestion overlap. Across the complete dataset, UAV altitude ranges from 15.0 to 173.1 m with a median of 88.1 m, while UAV-to-CS distance ranges from 5.0 to 800.0 m with a median of 90.7 m. Geographic coordinates, UAV trajectories, velocities, and waypoint sequences are not included in the released CSV; therefore, spatial and wireless context is represented using the available altitude, relative-distance, RSSI, signal-drift, and neighboring-congestion measurements.

The target variable, Attack_State, contains five classes: Normal (0), Low-Rate DDoS (1), High-Rate DDoS (2), Coordinated DDoS (3), and Application-Layer DDoS (4). Their distributions are 91,128 (82.94%), 6532 (5.95%), 4510 (4.10%), 3213 (2.92%), and 4489 (4.09%) observations, respectively. Within the released telemetry, Low-Rate DDoS is characterized by sustained traffic activity and increased session-initiation failures, High-Rate DDoS exhibits stronger packet-arrival and UDP-flood activity, Coordinated DDoS shows greater persistence and neighboring-congestion overlap, and Application-Layer DDoS is associated with increased API latency, authentication-queue length, and charging-authorization delay. The repository-provided Attack_State labels are used directly without manual relabeling.

The released CSV begins at the 10 min cyber-physical observation level and does not include the underlying packet/event stream, individual attack-generator identities, attack-source host identifiers, gateway identifiers, native geographic-zone identifiers, communication-technology labels, or attack-injection scripts. Consequently, packet-level attack generation, communication technology, gateway topology, and exact injection duration cannot be reconstructed from the released observations without introducing unsupported assumptions. Contiguous DDoS labels span one to three sampling intervals, corresponding to approximately 10–30 min of consecutive labeled observations; these intervals describe the released attack-state sequence rather than the original packet-level attack duration. Because the pre-aggregation event stream is unavailable, the low-level aggregation procedure used to construct each 10 min observation cannot be independently reconstructed. Each released record and its corresponding Attack_State label are therefore treated as the atomic observation in the experiments.

For leakage-controlled evaluation, all observations are ordered by timestamp before partitioning. The earliest 80% form the development set and the latest 20% form the independent chronological test set. The development set is further divided chronologically into training and validation subsets. A six-sample temporal embargo, corresponding to 60 min at the 10 min sampling interval and matching the temporal context R=6, is applied at the partition boundaries. Temporal windows are constructed independently within each partition so that no window crosses a training, validation, or test boundary. Normalization statistics, feature-selection thresholds, and class-rebalancing parameters are estimated exclusively from the training subset and then applied unchanged to the validation and test data. The validation subset is used for convergence monitoring, HHMAO-based hyperparameter selection, and model selection, whereas the independent test set is evaluated only after the final model configuration has been fixed.

Generalization is further assessed using charging-station-, EV-, and UAV-disjoint protocols, in which complete entity identities are assigned exclusively to either the development or evaluation partition. These protocols prevent the same physical entities from appearing on both sides of the evaluation and provide a stricter assessment of performance on previously unseen charging stations, EVs, and UAV monitoring entities. Table 2 summarizes the dataset composition, class distribution, UAV observation ranges, and evaluation protocol, while Table 3 presents the cyber-physical feature groups used by STAG-GuardNet.

The input variables are organized into two sensing domains for domain-specific analysis. Ground CS–EV telemetry includes network-traffic measurements, charging-station resource utilization, and EV communication variables, whereas UAV-assisted telemetry includes UAV altitude, UAV-to-CS distance, RSSI fluctuation, signal-drift index, and neighboring-CS congestion overlap. Temporal and contextual variables are recomputed from the variables available within each evaluated sensing configuration.

### 3.3. Data Preprocessing and Spatiotemporal Signal Conditioning

The CS–EV and UAV-assisted telemetry exhibit time-varying behavior across communication, charging, and attack conditions. Lightweight spatiotemporal conditioning separates slowly varying baseline behavior from short-term deviations before graph and temporal learning. Within each independently partitioned sequence, the *j*-th feature observed at time index *k* is organized into a sliding window of fixed temporal context; windows are never constructed across training, validation, or test boundaries. Each signal is decomposed into a baseline component and a short-term deviation component as follows:

(1) $$y_{j}\left(t\right)=b_{j}\left(t\right)+d_{j}\left(t\right),$$

The baseline $b_{j}\left(t\right)$ represents the slowly varying operating state of feature *j*, while $d_{j}\left(t\right)$ captures its short-term deviation. The baseline is updated recursively as

(2) $$b_{j}\left(t\right)=\lambda_{s}y_{j}\left(t\right)+\left(1-\lambda_{s}\right)b_{j}\left(t-1\right),$$

The smoothing coefficient $\lambda_{s}\in \left(0,1\right)$ controls the adaptation rate of the causal baseline. The deviation signal is then obtained as $d_{j}\left(t\right)=y_{j}\left(t\right)-b_{j}\left(t\right)$. Robust median–IQR scaling preserves transient deviations while reducing the influence of extreme values.

(3) $${\tilde{d}}_{j}\left(t\right)=\frac{d_{j}\left(t\right)-\mathrm{med}\left(d_{j}\right)}{\mathrm{IQR}(d_{j})+\epsilon },$$

med(·) and IQR(·) denote the median and interquartile range, respectively, while ϵ is a small constant that prevents division by zero. The final representation that has been conditioned consists of normalized deviations combined with baseline trends.

(4) $$X\left(t\right)={\left[{\tilde{d}}_{j}\left(t\right),b_{j}\left(t\right)\right]}_{j=1}^{F_{0}},$$

where $F_{0}$ denotes the number of input variables before feature filtering. The resulting representation preserves slowly varying operating behavior together with short-term deviations for subsequent graph and temporal learning.

To prevent target leakage, the repository-provided engineered trend, deviation, and persistence fields are not used directly. Their model inputs are recomputed causally within each partition from observable telemetry only. Let Q contain packet-arrival rate, SYN/ACK ratio, UDP-flood intensity, session-initiation failures, API latency, authentication-queue length, EV handshake retries, charging-authorization delay, message-retransmission rate, protocol-downgrade attempts, RSSI fluctuation, and neighboring-congestion overlap. For node *i*, the causal traffic-trend, baseline-deviation, and persistence scores are

(5) $$\begin{array}{cc} g_{i}\left(t\right)\; & =\frac{1}{\left|Q\right|}\sum_{q\in Q}\left|{\bar{x}}_{iq}\left(t\right)-{\bar{x}}_{iq}\left(t-1\right)\right|,\\ d_{i}\left(t\right)\; & =\frac{1}{\left|Q\right|}\sum_{q\in Q}\left|{\bar{x}}_{iq}\left(t\right)-{\bar{b}}_{iq}\left(t\right)\right|,\\ p_{i}\left(t\right)\; & =\rho_{p}p_{i}\left(t-1\right)+\left(1-\rho_{p}\right)d_{i}\left(t\right), \end{array}$$

where ${\bar{b}}_{iq}\left(t\right)$ is the causal baseline updated from observations available up to time *t*, and $\rho_{p}\in \left[0,1\right)$ controls persistence memory. Thus, $g_{i}\left(t\right)$ uses only observations at *t* and t−1, $d_{i}\left(t\right)$ uses the current observation and its recursively maintained past baseline, and $p_{i}\left(t\right)$ uses the current deviation and the previous persistence state. No Attack_State value, future observation t+h (h>0), or post-event information enters these computations. All states are initialized and updated separately within the training, validation, and test partitions.

### 3.4. Spatiotemporal Feature Encoding and Class Rebalancing

The conditioned CS–EV and UAV-assisted telemetry is transformed into a compact representation for spatio-temporal learning. Let V(t)=X(t) denote the conditioned feature vector at time *t*, where $V\left(t\right)\in R^{F}$ and *F* denotes the number of retained variables. Short-term changes in communication, infrastructure utilization, and wireless conditions are represented through the first-order difference

(6) Δ(t)=V(t)−V(t−1),

which captures changes between consecutive observations. The conditioned state and its short-term variation are then combined as

(7) $$H\left(t\right)=\left[V(t),\Delta (t)\right].$$

The combined representation preserves the current cyber-physical state and its short-term variation. A variance-based feature filter subsequently removes near-constant dimensions, with the dispersion of feature dimension *q* computed as

(8) $$\psi_{q}=\mathrm{Var}\;\left(H_{q}\left(n\right)\right),$$

Only feature dimensions satisfying $\psi_{q}>\kappa$ are retained, where κ is selected during hyperparameter optimization. The resulting representation is used for subsequent graph and temporal learning.

Class imbalance is handled through training-only class-conditional Gaussian augmentation in the encoded feature space. No separate trainable encoder or decoder is introduced; after conditioning and feature filtering, the retained representation $H\left(n\right)\in R^{P}$ itself forms the augmentation space, i.e., $z_{n}=H\left(n\right)$. For class *r*, only samples belonging to the training partition are used to estimate

(9) $$\nu_{r}=\frac{1}{N_{r}}\sum_{n:y_{n}=r}z_{n},\;\Lambda_{r}={\hat{\Sigma }}_{r}+\epsilon_{\Sigma }I,$$

where $N_{r}$ is the number of training samples in class *r*, ${\hat{\Sigma }}_{r}$ is its empirical covariance matrix, and $\epsilon_{\Sigma }=10^{-6}$ provides covariance regularization. Additional class-conditional encoded samples are generated as

(10) $${\tilde{z}}_{r,g}∼N\;\left(\nu_{r},\Lambda_{r}\right),\;g=1,\ldots ,G_{r}.$$

The Normal class is left unchanged, while the four DDoS classes are augmented to the size of the largest attack class in the training partition. Let $N_{\mathrm{atk}}^{\max}=\max_{r\in \{1,2,3,4\}}N_{r}$; the number of generated samples for class *r* is therefore $G_{r}=\max\left(0,N_{\mathrm{atk}}^{\max}-N_{r}\right)$. Because augmentation is performed directly in the encoded feature space consumed by STAG-GuardNet, the projection function is the identity, $P\left(\tilde{z}\right)=\tilde{z}$, and no separate back-projection network is required. Generated feature values are clipped to the corresponding ranges estimated from the training partition. All distribution statistics and generated samples are obtained exclusively from $D_{\mathrm{tr}}$; validation and test observations are never used for augmentation.

Class weighting addresses the remaining imbalance between the unchanged Normal class and the augmented DDoS classes. The weights used in Equation (23) are calculated from the post-augmentation training distribution as $w_{k}=N_{\mathrm{tr}}/\left(|C\right|N_{k})$ and remain fixed during optimization.

### 3.5. Spatio-Temporal Attack Graph Guard Network (STAG-GuardNet)

**Figure 2:**
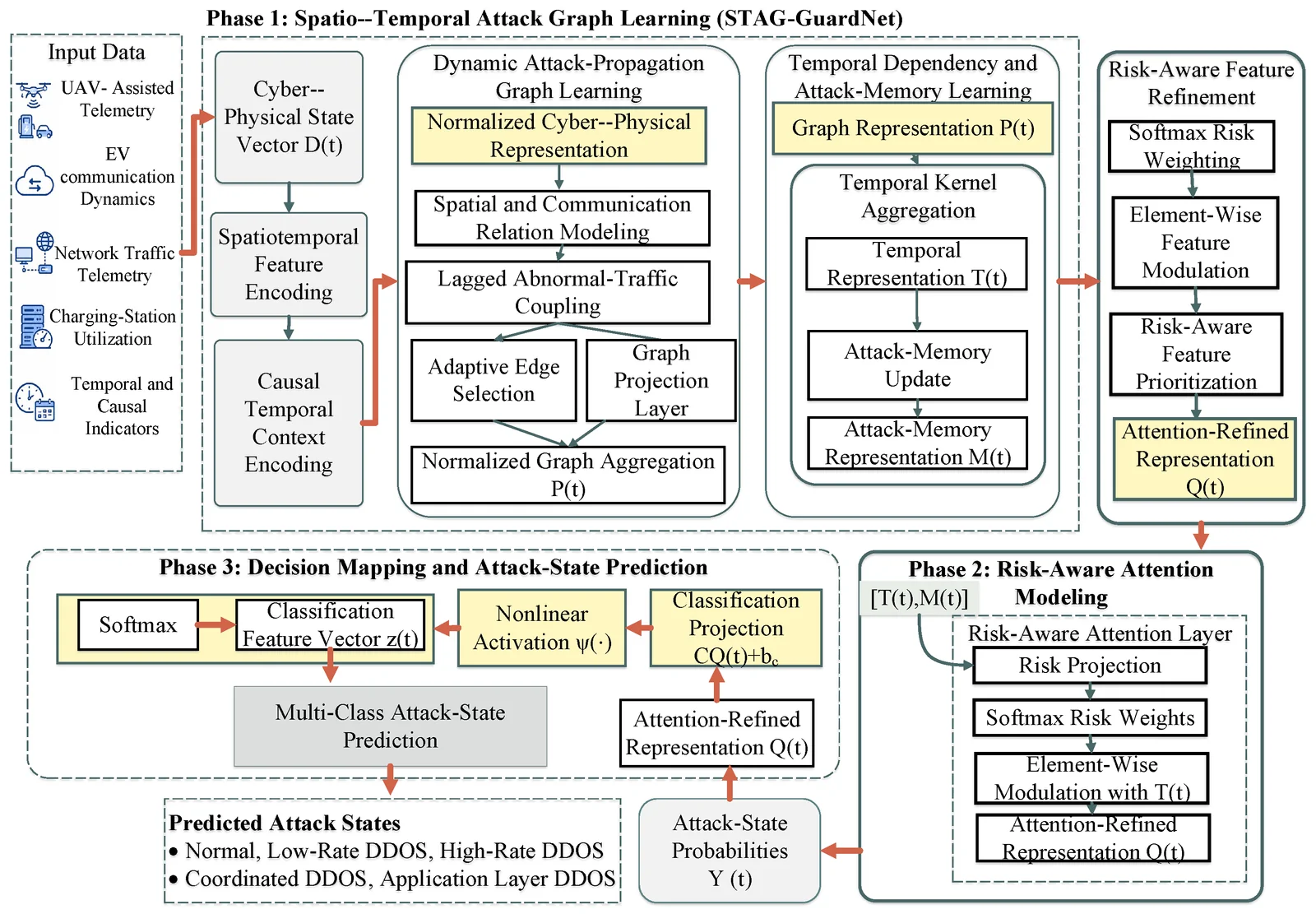
Architecture view of the proposed STAG-GuardNet framework.

After preprocessing, feature encoding, and class rebalancing, the cyber-physical state at time *t* is represented as

(11) $$D\left(t\right)=\left[D_{\mathrm{CS}}\left(t\right),D_{\mathrm{EV}}\left(t\right),D_{\mathrm{UAV}}\left(t\right)\right],$$

where $D_{\mathrm{CS}}\left(t\right)$ contains charging-station telemetry, $D_{\mathrm{EV}}\left(t\right)$ represents EV communication behavior, and $D_{\mathrm{UAV}}\left(t\right)$ contains UAV-assisted observations, including UAV-to-CS distance, wireless-signal variation, and congestion-related indicators. The resulting representation combines communication, infrastructure, and UAV-assisted information for spatiotemporal attack analysis. The STAG-GuardNet architecture is illustrated in Figure 2.

STAG-GuardNet constructs a directed telemetry-adaptive interaction graph, in which the set of charging-station-centered CS–EV nodes remains fixed for the evaluated infrastructure, while the retained directed edges and their weights are recomputed at each time step from the available telemetry. Spatial affinity, communication similarity, and lagged abnormal-traffic behavior determine these time-dependent relationships, while UAV telemetry provides spatial and wireless context to each node. Graph-specific features are first normalized to [0,1] using training-set statistics as

(12) $${\bar{x}}_{iq}\left(t\right)=\mathrm{clip}\;\left(\frac{x_{iq}\left(t\right)-x_{q}^{\min}}{x_{q}^{\max}-x_{q}^{\min}+\epsilon },0,1\right),$$

where $x_{q}^{\min}$ and $x_{q}^{\max}$ are obtained exclusively from the training data. Let $s_{i}\left(t\right)$ denote the normalized UAV-assisted spatial vector of node *i* and $c_{i}\left(t\right)$ its normalized communication-behavior vector. The spatial affinity and communication similarity are defined as

(13) $$\begin{array}{cc} S_{ij}\left(t\right)\; & =\exp\;\left(-\frac{∥s_{i}\left(t\right)-s_{j}{\left(t\right)∥}_{2}^{2}}{2\sigma_{s}^{2}+\epsilon }\right),\\ R_{ij}\left(t\right)\; & =\frac{c_{i}{\left(t\right)}^{⊤}c_{j}\left(t\right)}{∥c_{i}{\left(t\right)∥}_{2}{∥c_{j}\left(t\right)∥}_{2}+\epsilon }, \end{array}$$

where $\sigma_{s}$ is the median pairwise distance among the training spatial vectors. The abnormal-traffic coupling is constructed from the causal baseline-deviation score $d_{i}\left(t\right)$ defined in Section 3.2 as

(14) $$B_{ij}\left(t\right)=d_{i}\left(t-1\right)d_{j}\left(t\right).$$

Thus, $B_{ij}\left(t\right)$ uses only the source-node deviation available at t−1 and the destination-node telemetry available at the current decision time *t*. No Attack_State label, future observation t+h for h>0, or post-event information is used in its construction. The spatial term $S_{ij}\left(t\right)$ and communication term $R_{ij}\left(t\right)$ are likewise computed only from telemetry available at time *t*. Since the underlying quantities are normalized to [0,1], all three graph components are bounded within [0,1]. The one-step lag in $B_{ij}\left(t\right)$ provides directionality, so a strong relation i→j indicates that abnormal activity at node *i* precedes abnormal activity at node *j*. The combined edge score is

(15) $${\tilde{A}}_{ij}\left(t\right)=\omega_{s}S_{ij}\left(t\right)+\omega_{c}R_{ij}\left(t\right)+\omega_{a}B_{ij}\left(t\right),\;\omega_{s},\omega_{c},\omega_{a}\geq 0,\;\omega_{s}+\omega_{c}+\omega_{a}=1.$$

To obtain a sparse graph, outgoing candidate edges from node *i* are retained when their score exceeds the adaptive threshold $\tau_{i}\left(t\right)=\mu_{i}\left(t\right)+\sigma_{i}\left(t\right)$, where $\mu_{i}\left(t\right)$ and $\sigma_{i}\left(t\right)$ are the mean and standard deviation of its non-self edge scores. The neighborhood and final adjacency are defined as

(16) $$N_{i}\left(t\right)=\left\{j\neq i:{\tilde{A}}_{ij}\left(t\right)\geq \tau_{i}\left(t\right)\right\},\;A_{ij}\left(t\right)=\left\{\begin{array}{cc} {\tilde{A}}_{ij}\left(t\right), & j\in N_{i}\left(t\right),\\ 1, & i=j,\\ 0, & \mathrm{otherwise}. \end{array}\right.$$

At each time step, the neighborhood $N_{i}\left(t\right)$ is recomputed from the current edge scores and adaptive threshold. Consequently, an external edge may appear or disappear over time, and its weight may also change, while the charging-station-centered node identities remain fixed. If no external edge satisfies the threshold, the highest-scoring non-self neighbor is retained to prevent node isolation. Self-loops preserve the local cyber-physical state during message passing, while the lagged abnormality term allows $A_{ij}\left(t\right)\neq A_{ji}\left(t\right)$, yielding a directed telemetry-adaptive graph. Graph aggregation is then performed as

(17) $$P_{i}\left(t\right)=\frac{\sum_{j\in N_{i}\left(t\right)\cup \left\{i\right\}}A_{ij}\left(t\right)W_{g}D_{j}\left(t\right)}{\sum_{j\in N_{i}\left(t\right)\cup \left\{i\right\}}A_{ij}\left(t\right)+\epsilon },$$

where $W_{g}$ is a trainable graph projection matrix. This operation combines the local state with strongly related spatial, communication, and lagged abnormal-traffic states.

The framework subsequently maintains an attack-memory state to preserve persistent abnormal behavior:

(18) $$M\left(t\right)=\rho_{m}M\left(t-1\right)+\left(1-\rho_{m}\right)P\left(t\right),$$

where $\rho_{m}$ controls the attack-memory retention strength. The memory representation helps distinguish short-lived traffic disturbances from sustained or coordinated attack behavior.

Temporal dependencies are modeled over the preceding *R* states as

(19) $$T\left(t\right)=\sum_{r=0}^{R-1}U_{r}P\left(t-r\right),$$

where $U_{r}$ denotes a trainable temporal kernel and *R* defines the temporal context length.

A risk-aware attention mechanism then emphasizes states associated with abnormal communication activity, authentication delay, congestion, and spatially correlated behavior:

(20) $$Q\left(t\right)=T\left(t\right)\circ \mathrm{Softmax}\;\left(W_{r}\left[T\left(t\right),M\left(t\right)\right]+b_{r}\right),$$

where $W_{r}$ is a trainable transformation matrix, $b_{r}$ is the risk-attention bias vector, and ∘ denotes element-wise interaction.

The refined representation is mapped to the decision space as

(21) $$z\left(t\right)=\psi \;\left(CQ\left(t\right)+b_{c}\right),$$

where $b_{c}$ denotes the classification bias vector and ψ(·) is the nonlinear activation function. The final attack-state probabilities are obtained by

(22) $$\hat{y}\left(t\right)=\mathrm{Softmax}\;\left(z(t)\right).$$

Model parameters are learned using weighted cross-entropy to reduce the influence of class imbalance:

(23) $$L=-\frac{1}{N}\sum_{t=1}^{N}\sum_{k\in C}w_{k}y_{k}\left(t\right)\log{\hat{y}}_{k}\left(t\right),$$

where C={0,1,2,3,4} denotes the five attack-state classes and *N* is the number of training samples. The class weight is computed from the post-augmentation training distribution as $w_{k}=N/\left(|C\right|N_{k})$, where $N_{k}$ is the number of training observations belonging to class *k* after augmentation. The weights are fixed before model optimization. Latent augmentation therefore increases minority-class representation in the training data, whereas weighted cross-entropy modifies the loss contribution of the remaining class imbalance.

Algorithm 1 summarizes the complete training and prediction workflow. HHMAO forms the outer search procedure for selecting the hyperparameter configuration, whereas Adam updates the trainable STAG-GuardNet weights within each candidate evaluation through the forward pass and weighted classification loss. Candidate configurations are assessed on the validation partition, and the selected configuration is subsequently fixed for prediction on the independent test partition.

### 3.6. Hybrid Hawk–Manta Adaptive Optimization for Hyperparameter Tuning

HHMAO combines Harris Hawks Optimization (HHO) with Manta Ray Foraging Optimization (MRFO) for offline hyperparameter search [34,35], while Adam separately optimizes the trainable network weights. HHO provides broad candidate-space exploration during the first 60% of the search, after which MRFO refines promising configurations during the remaining iterations. The hyperparameter search vector is defined as

(24) $$\theta =[\lambda_{s},\kappa ,R,L_{g},p_{d},\rho_{m},\rho_{p},\omega_{s},\omega_{c},\omega_{a},\eta_{h},E_{0}],$$

Here, $\lambda_{s}$ is the signal-smoothing coefficient, κ is the feature-variance threshold, *R* is the temporal context length, $L_{g}$ is the graph depth, $p_{d}$ is the dropout probability, $\rho_{m}$ is the attack-memory retention coefficient, $\rho_{p}$ is the causal persistence coefficient, $\left(\omega_{s},\omega_{c},\omega_{a}\right)$ are the spatial, communication, and abnormal-traffic graph weights, $\eta_{h}$ is the HHMAO refinement coefficient, and $E_{0}$ is the initial HHO escaping-energy parameter. The graph weights are constrained to be non-negative and to sum to one.
**Algorithm 1** STAG-GuardNet Training and Prediction Framework**Require:** 
Training set $D_{\mathrm{tr}}$, validation set $D_{\mathrm{val}}$, test set $D_{\mathrm{te}}$, HHMAO population size *M*, iteration limit *I*, and candidate hyperparameter vector θ**Ensure:** 
Optimized configuration $\theta^{*}$ and predicted attack-state probabilities  1:Initialize HHMAO population $P={\left\{\theta^{\left(m\right)}\right\}}_{m=1}^{M}$  2:**for** i=1 to *I* **do**  3:   **for** m=1 to *M* **do**  4:     Configure STAG-GuardNet using candidate $\theta^{\left(m\right)}$ and initialize trainable network weights  5:     **for** each training epoch **do**  6:        **for** each mini-batch $B\subset D_{\mathrm{tr}}$ **do**  7:          Construct the telemetry-adaptive graph and compute graph aggregation P(t)  8:          Update attack memory M(t) and temporal representation T(t)  9:          Apply risk-aware attention and obtain predictions $\hat{y}\left(t\right)$10:          Compute weighted cross-entropy loss L11:          Update trainable network weights using Adam and backpropagation12:        **end for**13:     **end for**14:     Evaluate $\theta^{\left(m\right)}$ on $D_{\mathrm{val}}$ using the validation objective $F(\theta^{\left(m\right)})$15:   **end for**16:   Update candidate configurations using HHO exploration and MRFO refinement17:**end for**18:Select $\theta^{*}$ with the best validation objective and fix the corresponding STAG-GuardNet configuration19:Perform the forward pass on $D_{\mathrm{te}}$ using the fixed trained model without HHMAO updates20:**return **$\theta^{*}$ and test-set attack-state probabilities

Continuous parameters were initialized within their stated bounds, integer parameters were sampled from their discrete candidate sets, the graph coefficients were initialized on the probability simplex, and the regularization coefficient $\lambda_{r}=10^{-4}$ was fixed and excluded from the HHMAO search vector. HHMAO uses a deterministic exploration-refinement schedule over I=160 search iterations. HHO-based exploration is applied during the first 60% of the search, i.e., iterations $t\leq t_{s}$ with $t_{s}=\left\lfloor 0.60I\right\rfloor =96$, and MRFO-based refinement is applied during the remaining iterations. Each search run uses a population of M=40 candidate configurations. After every update, continuous variables are clipped to their bounds, *R* and $L_{g}$ are rounded to the nearest admissible integer after clipping, and $\left(\omega_{s},\omega_{c},\omega_{a}\right)$ is projected onto the non-negative unit simplex. A newly generated candidate replaces its previous configuration only when it yields a lower validation objective F; the global best is updated using the same criterion. Random quantities required by HHO and MRFO are sampled independently from their stated uniform distributions. Five independent search runs are performed using seeds {11,23,37,51,73}, with the data partitions held unchanged across runs. The reported search comparison summarizes performance across these repeated runs. For the reported experiments, iteration t=1 evaluates the initialized population and the search terminates after exactly I=160 population evaluations, giving MI=40×160=6400 candidate-model evaluations per search run; no search-level early stopping is applied.

The HHMAO population is initialized as

(25) $$P=\{\theta^{\left(1\right)},\theta^{\left(2\right)},\ldots ,\theta^{\left(M\right)}\},$$

where *M* denotes the population size.

Each candidate configuration is evaluated using the validation objective

(26) $$F\left(\theta^{\left(m\right)}\right)=L_{\mathrm{val}}\left(\theta^{\left(m\right)}\right)+\lambda_{r}{∥\theta^{\left(m\right)}∥}_{2},$$

where $L_{\mathrm{val}}$ is the validation loss and $\lambda_{r}$ is the validation-objective regularization coefficient, distinct from the signal-smoothing coefficient $\lambda_{s}$.

During HHO exploration, candidate configurations are updated as

(27) $$\theta_{t+1}^{\left(m\right)}=\theta_{t}^{\mathrm{best}}-E\left|J\theta_{t}^{\mathrm{best}}-\theta_{t}^{\left(m\right)}\right|,$$

where $\theta_{t}^{\mathrm{best}}$ denotes the current best solution, *E* is the iteration-dependent escaping-energy coefficient initialized from $E_{0}$ and adaptively decayed during the search, and *J* denotes the random jump strength.

During the MRFO refinement phase, chain-foraging updates move candidate configurations toward promising regions of the search space as

(28) $$\theta_{t+1}^{\left(m\right)}=\theta_{t}^{\left(m\right)}+\eta_{h}\left(\theta_{t}^{\mathrm{best}}-\theta_{t}^{\left(m\right)}\right),$$

where $\eta_{h}$ controls the HHMAO local refinement rate.

During the MRFO refinement stage, candidate configurations are updated using the chain- and cyclone-foraging operators defined in the original MRFO formulation [35]. Following each update, continuous variables are clipped to their search bounds, integer-valued parameters are repaired to the nearest admissible values, and the graph-weight coefficients are projected onto the non-negative unit simplex.

The HHO and MRFO stages follow the switching schedule defined in Algorithm 2. HHO explores the search space during the first 96 iterations, after which MRFO refines the population around promising configurations for the remaining 64 iterations. Candidate replacement follows a greedy validation-objective rule, and each search run terminates after exactly 160 population evaluations. With a population of 40 candidates, each run therefore performs 6400 candidate-model evaluations. Five independent search runs are conducted, corresponding to a maximum of 32,000 candidate evaluations across the complete HHMAO search experiment.
**Algorithm 2** Reproducible HHMAO Hyperparameter Search**Require:** 
Search bounds in Table 4, population size M=40, iterations I=160, switch iteration $t_{s}=96$, training set $D_{\mathrm{tr}}$, validation set $D_{\mathrm{val}}$, seed *s***Ensure:** 
Best hyperparameter vector $\theta^{*}$  1:Set random generators using seed *s*  2:Initialize *M* candidate vectors within the specified search bounds  3:Repair integer variables and project graph weights onto the unit simplex  4:Evaluate the initialized candidates using F and set $\theta^{*}$ to the best candidate  5:**for **t=2 to *I* **do**  6:   **for** m=1 to *M* **do**  7:     **if** $t\leq t_{s}$ **then**  8:        Generate $\theta^{\prime }$ using the HHO exploration update  9:     **else**10:        Generate $\theta^{\prime }$ using MRFO chain/cyclone refinement11:     **end if**12:     Clip continuous variables to their search bounds13:     Round *R* and $L_{g}$ to the nearest admissible integers14:     Project $\left(\omega_{s},\omega_{c},\omega_{a}\right)$ onto the non-negative unit simplex15:     Train STAG-GuardNet on $D_{\mathrm{tr}}$ using Adam and evaluate $F(\theta^{\prime })$ on $D_{\mathrm{val}}$16:     **if** $F\left(\theta^{\prime }\right)<F\left(\theta^{\left(m\right)}\right)$ **then**17:        $\theta^{\left(m\right)}\leftarrow \theta^{\prime }$18:     **end if**19:     **if** $F\left(\theta^{\left(m\right)}\right)<F\left(\theta^{*}\right)$ **then**20:        $\theta^{*}\leftarrow \theta^{\left(m\right)}$21:     **end if**22:   **end for**23:**end for**24:**return **$\theta^{*}$

HHMAO performs population-based hyperparameter search with computational complexity governed by the population size *M*, number of iterations *I*, search-space dimension *d*, and candidate-evaluation cost $C_{\mathrm{val}}$. The parameter-update operations require approximately O(IMd) complexity, while the overall search cost is dominated by candidate evaluation and can be expressed as $O\;\left(IM(C_{\mathrm{val}}+d)\right)$. Hyperparameter optimization is performed offline before final model training and deployment. The HHMAO search therefore contributes additional model-development cost beyond the final 160-epoch training run. Wall-clock and GPU-energy measurements for the complete historical hyperparameter-search process were not retained consistently across HHMAO and all comparison methods; consequently, the deployment-energy analysis below is restricted to the final configured-model training and inference stages and is not interpreted as an end-to-end optimization-energy comparison.

**Table 4:** HHMAO search space and selected STAG-GuardNet hyperparameters.

Parameter	Type	Search Bounds	Initialization	Selected Value
$\lambda_{s}$	Continuous	[0.05,0.50]	Uniform	0.20
κ	Continuous	$\left[10^{-5},\;10^{-2}\right]$	Log-uniform	$10^{-3}$
*R*	Integer	{3,…,12}	Discrete uniform	6
$L_{g}$	Integer	{1,…,5}	Discrete uniform	3
$p_{d}$	Continuous	[0.10,0.50]	Uniform	0.25
$\rho_{m}$	Continuous	[0.50,0.95]	Uniform	0.85
$\rho_{p}$	Continuous	[0.50,0.95]	Uniform	0.80
$\left(\omega_{s},\omega_{c},\omega_{a}\right)$	Constrained continuous	${\left[0,1\right]}^{3},\;\sum \omega =1$	Dirichlet (1,1,1)	(0.30,0.30,0.40)
$\eta_{h}$	Continuous	[0.10,0.60]	Uniform	0.35
$E_{0}$	Continuous	[0.50,2.00]	Uniform	1.50
$\lambda_{r}$	Fixed continuous	Not searched	Fixed	$1\times 10^{-4}$

### 3.7. Performance Evaluation

Classification performance is evaluated using accuracy, support-weighted precision, support-weighted recall, and support-weighted F1-score to characterize overall multi-class prediction performance under class imbalance [36]. Macro F1-score is additionally reported to provide an equal-weight assessment across all classes. Attack-focused sensitivity is summarized using the Attack Awareness Score (AAS), which corresponds to macro-average recall calculated over the four DDoS classes while excluding the Normal class:

(29) $$\mathrm{AAS}=\frac{1}{|C_{\mathrm{atk}}|}\sum_{k\in C_{\mathrm{atk}}}\frac{T_{k}}{T_{k}+M_{k}},$$

In Equation (29), $C_{\mathrm{atk}}$ contains the four DDoS classes, $T_{k}$ denotes correctly classified samples of attack class *k*, and $M_{k}$ denotes samples of that class assigned to another label. AAS therefore represents attack-class macro recall. The corresponding class recalls are 93.17% for Low-Rate DDoS, 89.19% for High-Rate DDoS, 88.62% for Coordinated DDoS, and 91.47% for Application-Layer DDoS, producing an AAS of 90.61%. The Normal class is excluded so that the measure summarizes sensitivity across the attack categories independently of the dominant Normal-class support.

## 4. Experimental Results and Discussion

The STAG-GuardNet framework is evaluated for DDoS and intrusion-detection performance across the primary EV-charging dataset and three established IoT security benchmarks. The experiments assess multi-dataset applicability rather than zero-shot cross-domain transfer. HSEC-DDoS, ToN_IoT, Edge-IIoTset, and X-IIoTID are processed, trained, validated, and tested independently, and model parameters learned from one dataset are not transferred to another dataset. Table 5 presents the training configuration, architectural parameters, and optimization settings.

The experimental evaluation reports accuracy, weighted precision, weighted recall, weighted F1-score, macro F1-score, and AAS. The weighted metrics summarize aggregate performance while accounting for class support, whereas the macro F1-score gives equal importance to all classes. AAS is reported as attack-class macro recall over the four DDoS categories and provides an attack-focused view of detection sensitivity under the imbalanced class distribution.

Table 5 summarizes the training, architectural, and hyperparameter-search settings. Adam with cosine learning-rate decay optimizes the trainable model weights, while weight decay and gradient clipping stabilize training. STAG-GuardNet combines telemetry-adaptive graph aggregation, temporal dependency learning, attack-memory encoding, and risk-aware attention. HHMAO is applied separately for hyperparameter selection, while robust scaling and training-only Gaussian augmentation support data conditioning and class balancing. The incorporation of cosine learning-rate scheduling together with regularization mechanisms and gradient stabilization strategies enabled the system to achieve better generalization performance across distributed charging infrastructures because these methods successfully reduced overfitting issues. The architectural design supports the successful combination of telemetry-adaptive graph aggregation with temporal dependency learning, attack-memory encoding, and adaptive risk-aware attention modeling to enable DDoS attack detection through coordinated efforts. The Hybrid Hawk–Manta Adaptive Optimizer (HHMAO) provides better convergence stability because it achieves both exploration and exploitation balance during the parameter optimization process. The combination of robust normalization with latent-space class balancing methods enables learning stability across multiple traffic conditions, infrastructure states, and cyber-physical attack scenarios.

**Figure 3:**
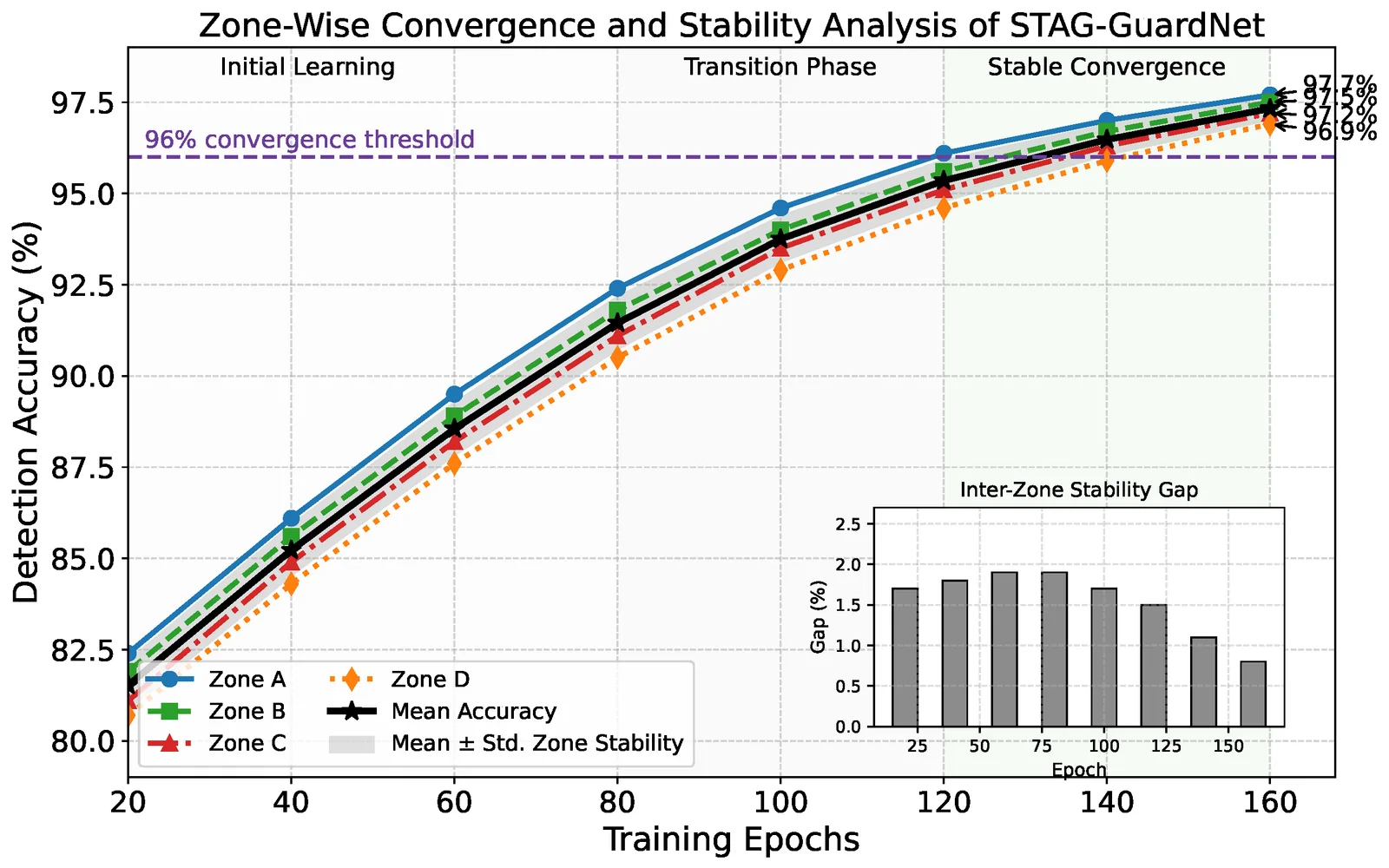
Convergence and stability analysis of STAG-GuardNet across repeated training runs.

**Figure 4:**
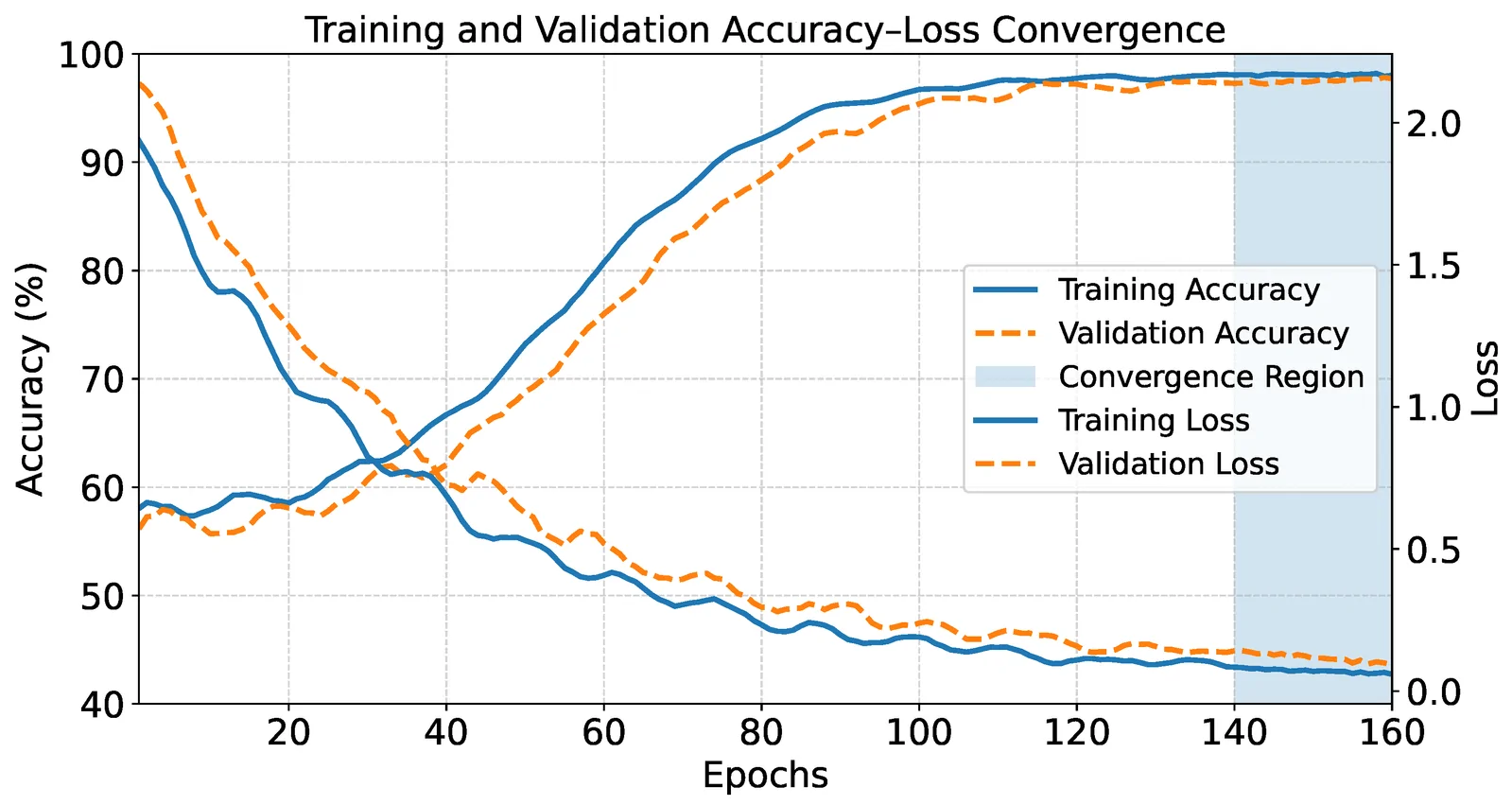
Training and validation accuracy-loss convergence of STAG-GuardNet over 160 epochs. Accuracy progressively increases while loss decreases for both training and validation, clearly distinguishing the corresponding convergence trends despite the similar line colors. The shaded region indicates the convergence stage after epoch 140.

Figure 3 demonstrates how STAG-GuardNet achieves convergence results through its operation in different charging station zones that experience diverse traffic patterns. The framework demonstrates stable and consistent learning performance across all regions while maintaining robust detection capability under varying communication loads and attack behaviors. The inter-zone convergence trends indicate that the proposed spatiotemporal attack-graph learning strategy effectively captures regional traffic dependencies and coordinated attack propagation patterns across distributed EV charging infrastructures. Figure 4 presents the training and validation accuracy and loss trajectories across 160 epochs. Both curves show progressive accuracy improvement and decreasing loss, with stable convergence after approximately epoch 140. The limited separation between the training and validation trajectories indicates stable model development without substantial overfitting. The independent chronological test partition is excluded from epoch-wise monitoring and is evaluated only after the final model configuration is fixed.

HSEC-DDoS is evaluated using a chronological partition, in which the earliest 80% of observations form the development set and the latest 20% form the independent test set. The development portion is further divided chronologically into training and validation subsets, with a 60 min embargo applied at partition boundaries. ToN_IoT, Edge-IIoTset, and X-IIoTID are evaluated independently using their corresponding training, validation, and test partitions. The literature-based comparative methods are reimplemented according to the architectural descriptions, training settings, and hyperparameter configurations provided in their corresponding publications, with input and output dimensions adapted where required for the evaluated datasets. The remaining comparative architectures are implemented directly for the datasets considered in this study. Within each dataset, STAG-GuardNet and all comparative methods use the same training, validation, and test observations, and the reported results are obtained from the present experimental evaluation rather than copied from the original publications. Data preparation is performed independently for each dataset, with normalization statistics, feature-selection parameters, and class-balancing information estimated exclusively from the corresponding training partition. Input features are provided according to the requirements of each architecture while preserving the same available cyber-physical observations, and output classes are mapped to the target classes of the corresponding dataset. Architecture-specific hyperparameters follow the published configurations where applicable and are selected using the validation partition, while the independent test partition remains excluded from model and hyperparameter selection.

Performance variability is assessed over 10 independent runs using the fixed random seeds {11,23,37,51,73,89,101,127,149,173}. Under each seed, the comparative methods use the same data partitions, while model initialization, minibatch ordering, and stochastic training operations vary across runs. Accuracy and weighted F1-score are reported as mean ± standard deviation. Statistical comparison on HSEC-DDoS uses paired weighted F1-score observations obtained from the corresponding runs. A two-sided Wilcoxon signed-rank test evaluates the paired differences, Cohen’s *d* quantifies practical effect size, and 95% confidence intervals characterize uncertainty in the mean weighted F1-score differences. Benjamini–Hochberg correction is applied across the multiple baseline comparisons, with an adjusted p<0.05 considered statistically significant.

Table 6 shows that STAG-GuardNet achieves the highest mean accuracy and weighted F1-score across the four independently evaluated datasets. On HSEC-DDoS, STAG-GuardNet achieves 97.7% accuracy and a 97.7% weighted F1-score, compared with 91.2% accuracy and a 90.9% weighted F1-score for BiRAGNN, the closest graph-recurrent comparison. BiRAGNN demonstrates that attentive graph modeling combined with recurrent learning can capture important spatial and sequential attack characteristics, while the additional telemetry-dependent graph updates, attack-memory representation, risk-aware feature interaction, and UAV-assisted context in STAG-GuardNet provide a richer representation of changing communication relationships and coordinated attack evolution. Strong performance is also maintained on ToN_IoT, Edge-IIoTset, and X-IIoTID, with comparatively small standard deviations across the repeated evaluations.

Table 7 summarizes the paired statistical analysis and shows consistent weighted F1-score improvements across the comparative methods. The mean differences range from 6.80 to 11.50 percentage points, and all 95% confidence intervals remain above zero. The paired Wilcoxon signed-rank probabilities range from 0.020 to 0.036, while the Benjamini–Hochberg-adjusted values remain below the 0.05 significance threshold after accounting for the 14 simultaneous comparisons. Cohen’s *d* ranges from 1.03 to 1.36, indicating large practical effects in addition to the observed statistical differences. The combined confidence-interval, significance, and effect-size results indicate that the performance improvements are consistent across repeated evaluations and are not explained solely by individual model initialization.

The component ablations use the same 10 fixed-seed runs, chronological partitions, and training settings as the complete model. Each configuration changes only the indicated component while retaining the remaining framework unchanged. The analysis complements the graph-recurrent comparison by isolating the roles of telemetry-dependent graph construction, temporal modeling, attack-memory encoding, and risk-aware attention within STAG-GuardNet. For the graph analysis, spatial affinity $S_{ij}$, communication similarity $R_{ij}$, and lagged abnormal-traffic coupling $B_{ij}$ are removed individually. The fixed-adjacency configuration constructs the adjacency matrix once from the training-period edge scores and retains it during training, validation, and testing, whereas the telemetry-adaptive configuration recomputes the retained edges and their weights from the observations available at each time step. Results are reported as mean ± standard deviation across the repeated runs.

Table 8 shows how the individual components contribute to the final representation learned by STAG-GuardNet. Among the graph components, removing the lagged abnormal-traffic coupling $B_{ij}$ produces the largest reduction, followed by spatial affinity $S_{ij}$ and communication similarity $R_{ij}$. Eliminating graph aggregation entirely causes a further decrease, confirming the importance of modeling relationships among distributed charging station-centered states. The fixed-adjacency configuration also achieves a substantially lower weighted F1-score than the telemetry-dependent telemetry-adaptive graph, indicating that evolving communication and attack relationships are not represented as effectively by fixed connectivity. The temporal components provide complementary information to the graph representation, with the largest degradation observed when temporal dependency learning is removed, followed by the removal of attack-memory encoding and risk-aware attention. Overall, these results show that STAG-GuardNet benefits from the combined modeling of changing graph relationships, temporal attack evolution, persistent abnormal behavior, and UAV-assisted cyber-physical context.

Table 9 shows the effect of the available sensing information on attack-state classification. CS–EV telemetry alone preserves the principal network and infrastructure attack indicators, while UAV-assisted telemetry alone provides a more limited representation of the underlying communication behavior. Combining the ground and UAV-assisted domains produces the strongest overall performance and attack-class recall. Performance remains higher than the ground-only configuration when UAV observations are partially degraded, indicating that the remaining aerial measurements continue to provide complementary spatial and wireless information.

Table 10 compares the individual and combined effects of the imbalance-handling strategies under the same chronological evaluation protocol. Class weighting, focal loss, and Gaussian augmentation progressively improve accuracy and weighted F1-score relative to training without explicit imbalance correction. The combination of Gaussian augmentation and class weighting provides the strongest performance, indicating that data-level augmentation and loss-level weighting provide complementary support for learning from the imbalanced attack-state distribution.

Post hoc model interpretation is performed using SHapley Additive exPlanations (SHAP) with the model-agnostic Kernel SHAP estimator applied to the STAG-GuardNet prediction pipeline. The background set contains 250 class-stratified training windows, while 500 observations from the independent test set are explained using 100 samples from each of the five attack-state classes. Each explained observation retains the six-step temporal context used by STAG-GuardNet. SHAP values are computed separately for each softmax output and aggregated across the six temporal positions using mean absolute attribution, yielding class-specific contributions of the original telemetry variables. Attribution stability is assessed over 10 class-stratified resamplings of the explained test subset using feature-rank consistency and top-feature overlap. These explanations characterize associations learned by the model and are not interpreted as causal evidence.

**Figure 5:**
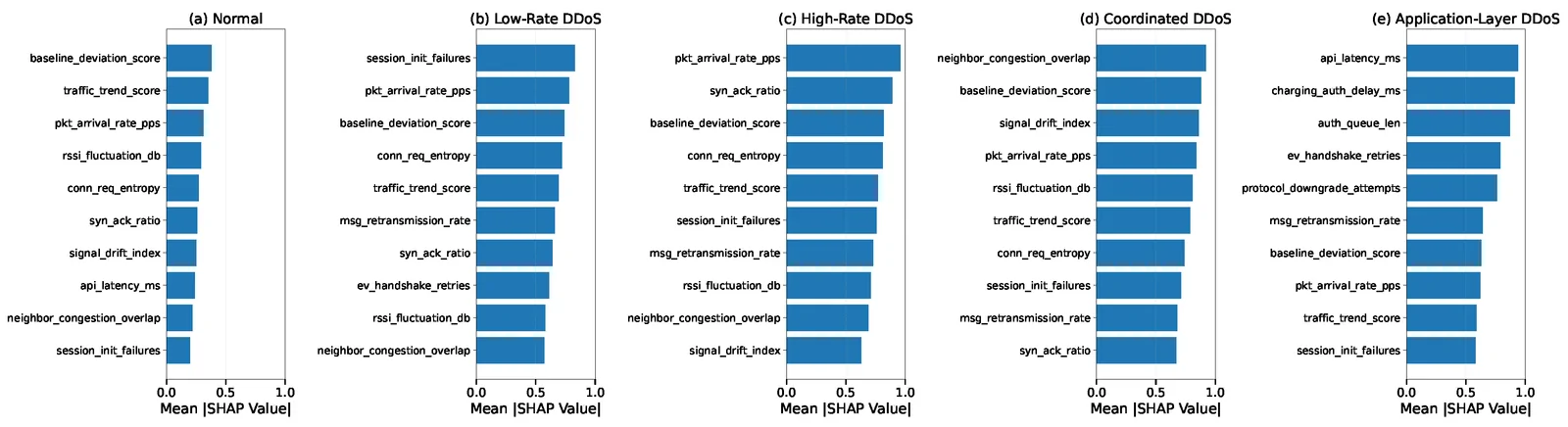
Class-specific mean absolute SHAP feature attributions across the five attack-state classes.

Figure 5 presents the class-specific feature attributions for Normal, Low-Rate DDoS, High-Rate DDoS, Coordinated DDoS, and Application-Layer DDoS predictions. Network-traffic and communication-delay variables contribute strongly to the DDoS classifications, while UAV-assisted spatial and wireless measurements provide complementary information for several attack classes. The repeated attribution analysis shows that the dominant feature rankings remain consistent across the class-stratified resamplings, indicating stable post hoc explanations for the evaluated test observations.

**Figure 6:**
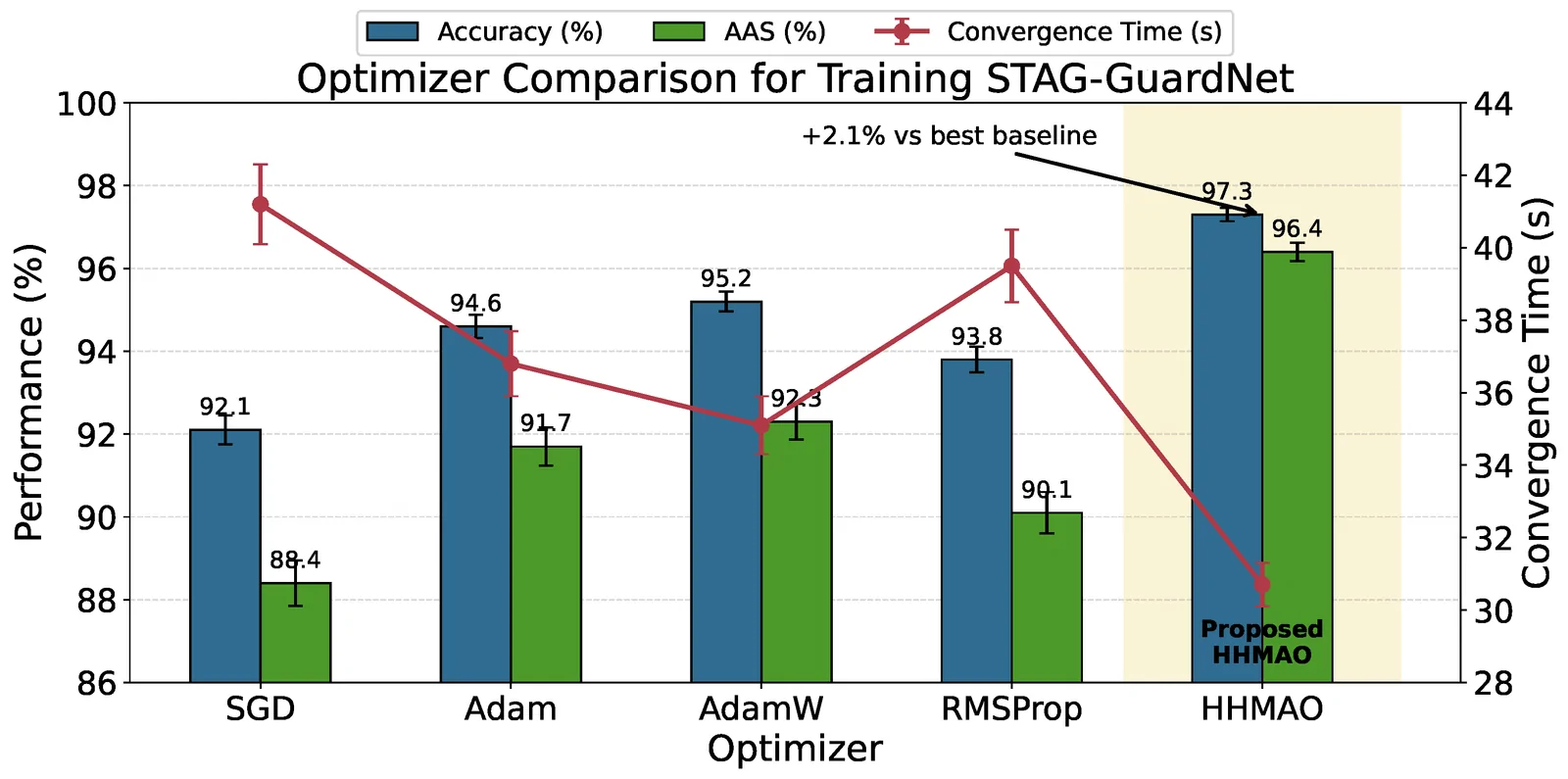
Comparison of hyperparameter-search methods for STAG-GuardNet with Adam fixed as the weight optimizer.

Hyperparameter-search performance is evaluated by comparing Random Search, Bayesian Optimization, HHO, MRFO, and HHMAO while keeping Adam fixed as the weight optimizer, as shown in Figure 6. The STAG-GuardNet architecture, chronological training-validation partition, hyperparameter search space, training configuration, and candidate-evaluation budget are held constant across all search methods. This controlled comparison isolates the effect of the hyperparameter-search strategy from gradient-based neural-network weight optimization.

Robustness is evaluated at three severity levels using the frozen model without retraining or fine-tuning. Dirichlet traffic heterogeneity is used only to construct centralized evaluation subsets with different attack-class proportions, using α∈{1.0,0.5,0.1}; lower α produces stronger class-distribution skew and does not represent federated clients. Missing telemetry is generated by independently masking 10%, 20%, or 30% of test-set feature entries and replacing them with the corresponding training-set medians. Gaussian corruption is applied to 20% of randomly selected numerical test entries as ${\tilde{x}}_{j}=x_{j}+\epsilon_{j}$, where $\epsilon_{j}∼N\left(0,{\left(\eta \sigma_{j,\mathrm{tr}}\right)}^{2}\right)$, $\sigma_{j,\mathrm{tr}}$ is the training-set standard deviation of feature *j*, and η∈{0.05,0.10,0.20} defines mild, moderate, and severe noise. Missingness and noise locations are sampled independently of the attack labels.

Charging-node outages are evaluated by randomly removing 10%, 20%, or 30% of charging-station nodes at inference time, independently of attack class. For each affected time step, the failed nodes and their incident edges are removed, and the spatial affinity $S_{ij}\left(t\right)$, communication similarity $R_{ij}\left(t\right)$, abnormal-traffic coupling $B_{ij}\left(t\right)$, adaptive thresholds, and final adjacency matrix are recomputed using only the remaining active nodes. Thus, all robustness conditions represent test-time perturbations of the same trained STAG-GuardNet model.

Table 11 summarizes the robustness of STAG-GuardNet under representative class-distribution skew, missing telemetry, Gaussian noise, and charging-node outage conditions. The trained model retains high accuracy and weighted F1-score across all evaluated perturbations without retraining or fine-tuning. Dirichlet skew evaluates sensitivity to a strongly imbalanced attack-class distribution, missing telemetry represents incomplete observations, Gaussian noise evaluates corrupted numerical measurements, and charging-node outage represents partial infrastructure unavailability. The results indicate that STAG-GuardNet maintains stable attack-state classification performance under heterogeneous and degraded cyber-physical observations.

**Figure 7:**
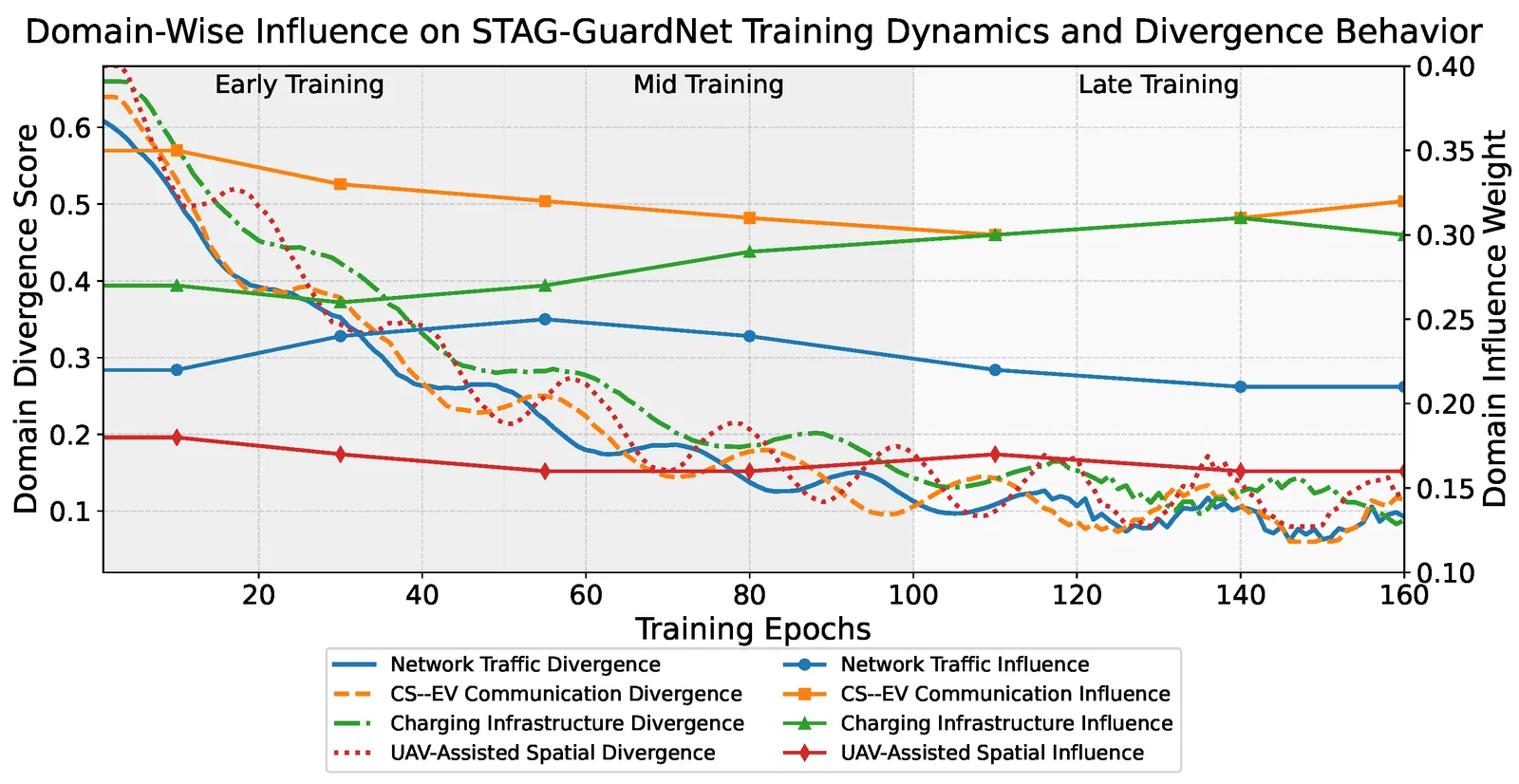
Domain-wise influence on STAG-GuardNet training dynamics and divergence behavior.

Figure 7 shows how the relative influence of the monitored feature domains changes during STAG-GuardNet training. The early learning phase is mainly affected by network traffic and CS-to-EV communications until charging infrastructure and UAV-assisted spatial and wireless features start to have greater impact during the training stabilization phase. The analysis indicates that STAG-GuardNet combines complementary information from multiple cyber-physical feature domains rather than relying on a single input group. The domain-aware learning method improves DDoS behavior detection accuracy for distributed smart charging systems that implement coordinated attacks.

Computational profiling is performed on a single NVIDIA GeForce RTX 4060 Laptop GPU with 8 GB VRAM using Python 3.14.7, PyTorch 2.13.0, CUDA 13.2, and cuDNN 9.25.1 under FP32 precision. Model training uses a batch size of 64, and the elapsed GPU execution time is measured for each training epoch across the 160-epoch training process. Inference latency is evaluated with batch size 1 after 100 warm-up forward passes, followed by 1000 independent test samples with GPU synchronization immediately before and after each timed interval. FLOPs correspond to the operations required for a single forward pass of one input sample, while memory consumption represents the peak GPU memory allocated during inference. GPU power is sampled through the NVIDIA Management Library (NVML) and integrated over execution time to determine training and inference energy. The same hardware environment, numerical precision, batch configuration, and profiling procedure are used for STAG-GuardNet and the comparative models. The computational profile covers model training and inference on the processing node, while UAV sensing, wireless transmission, gateway communication, and other network-level operations belong to the communication infrastructure outside the model-execution pipeline.

Table 12 summarizes the training time, parameter count, memory footprint, FLOPs, and inference latency of STAG-GuardNet and the comparative methods. Training and inference energy are obtained from the GPU power measurements described above. The reported training energy corresponds to the final configured 160-epoch model-training run, while inference energy is measured independently in mWh per test sample. Hyperparameter-search energy is not included because the tuning stages of HHMAO and the comparison methods were not profiled under a common wall-clock and GPU-energy measurement protocol. The reported values therefore characterize final-model computational cost rather than the total energy required for model development and hyperparameter optimization.

Table 13 shows that the final STAG-GuardNet training run consumes 227.2 Wh over 160 epochs and requires 0.031 mWh per sample during inference. The comparison models are evaluated using the same final-model profiling procedure. These measurements represent computational requirements after the model configuration has been fixed and do not include the preceding hyperparameter-search cost; therefore, they are not interpreted as an end-to-end optimization-energy comparison.

**Figure 8:**
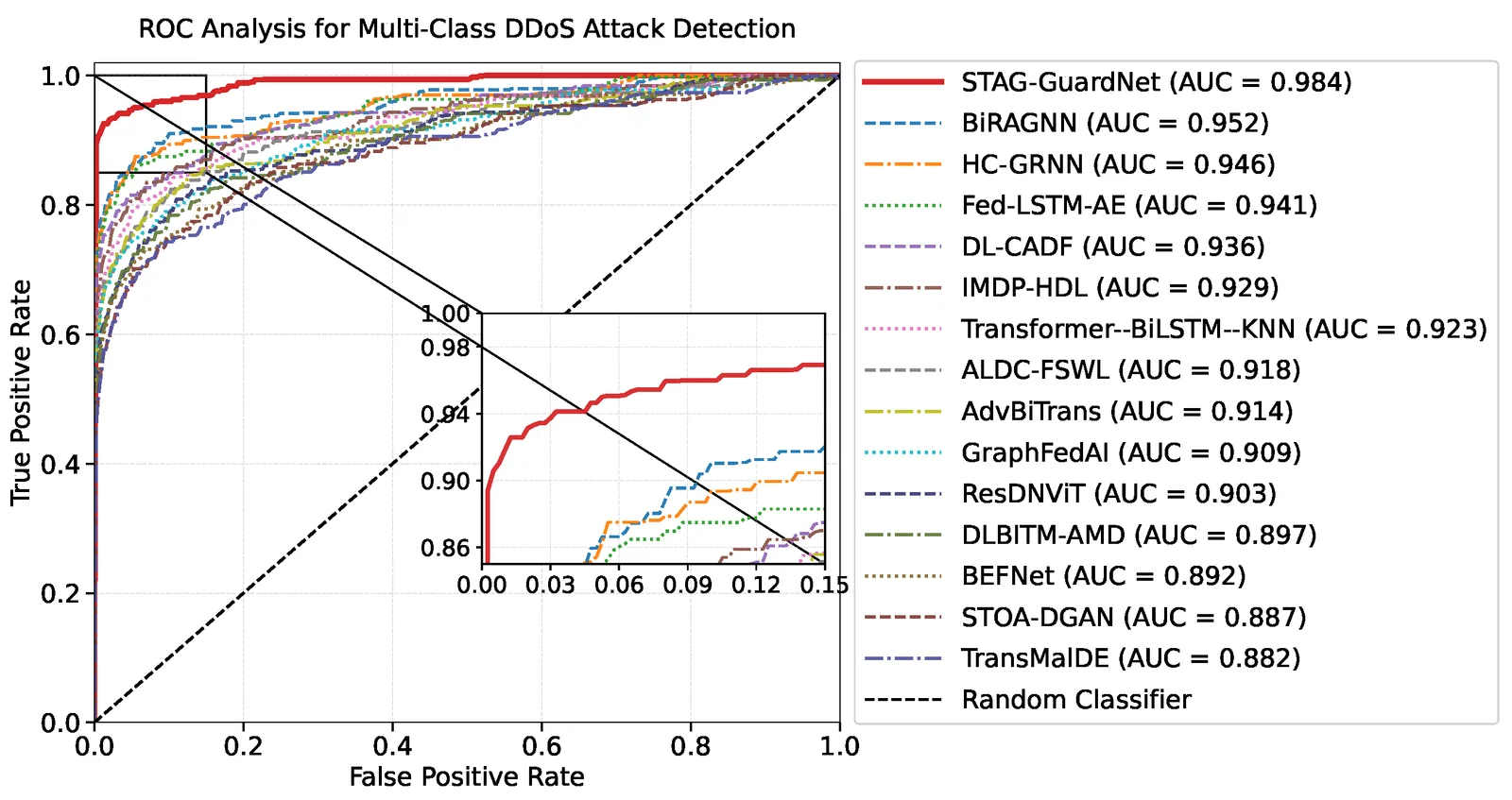
ROC comparison of STAG-GuardNet and baseline models with a zoomed inset highlighting the high-performance region (low FPR).

Figure 8 presents the ROC comparison between STAG-GuardNet and the selected baseline models.

**Figure 9:**
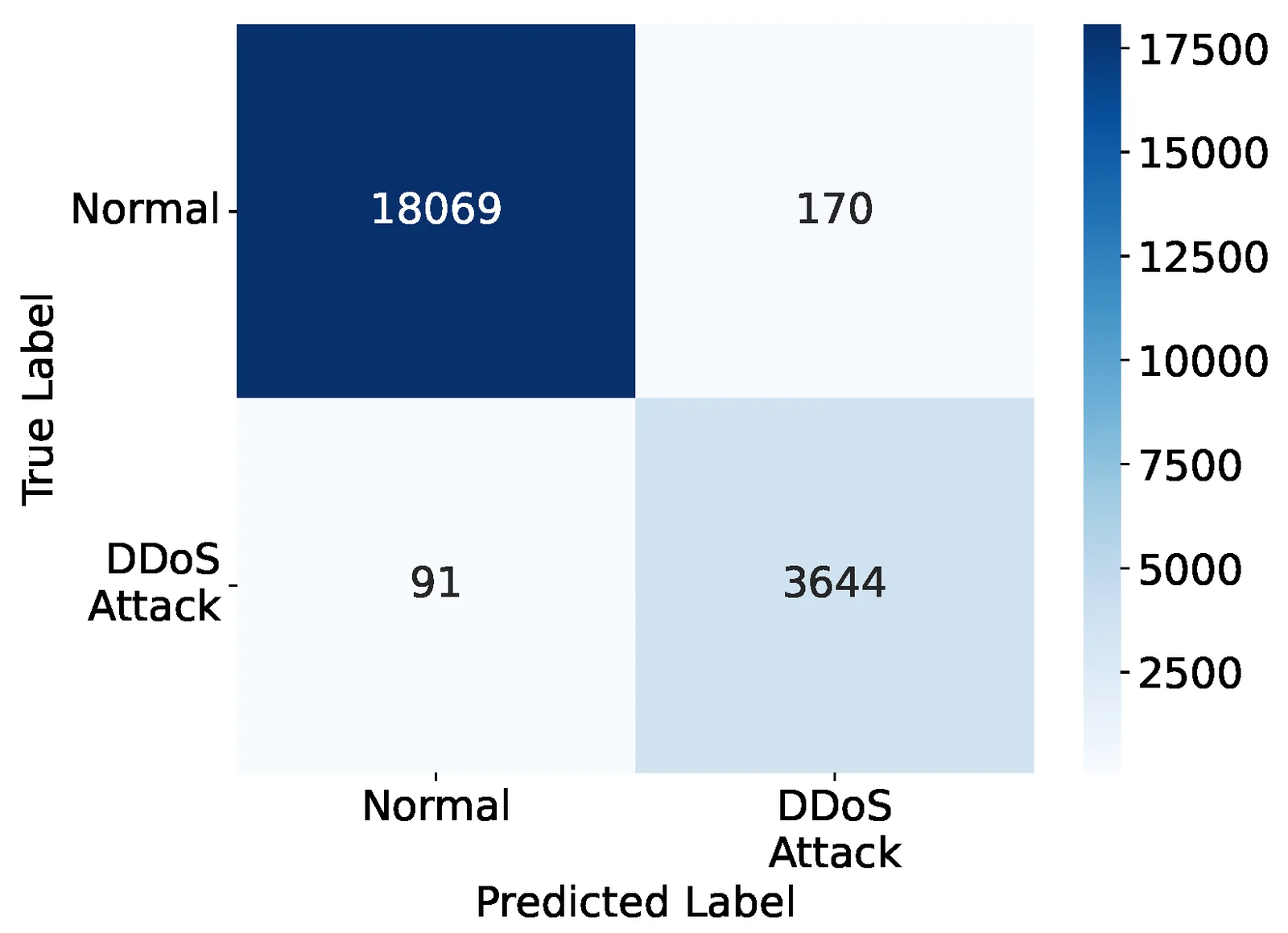
Binary confusion matrix for DDoS detection.

**Figure 10:**
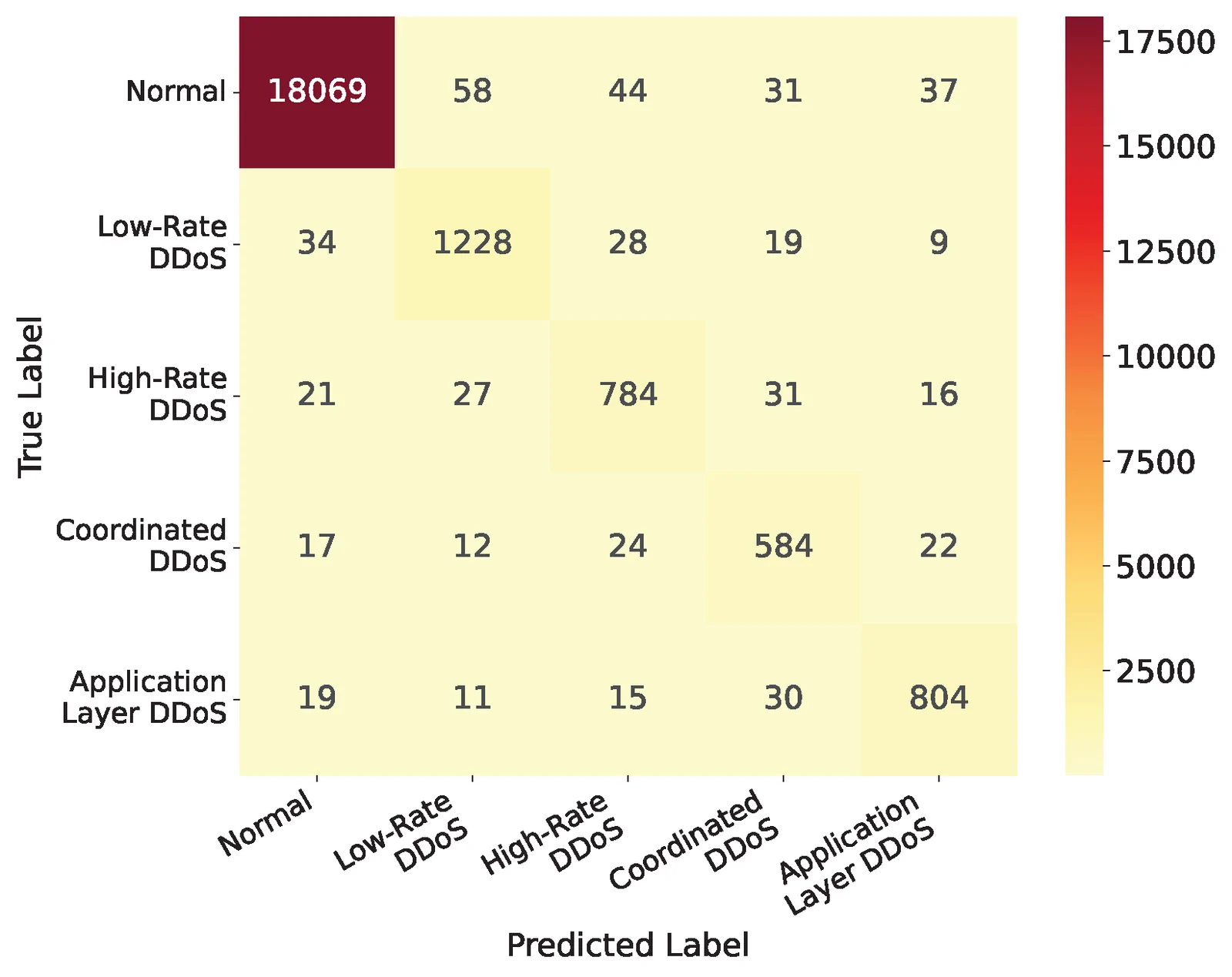
Five-class confusion matrix for DDoS attack-state classification.

All final classification metrics and confusion matrices are generated from a single frozen prediction file for the 21,974-record chronological test set using one common evaluation script. The binary confusion matrix in Figure 9, obtained by collapsing the four attack categories into a single DDoS class, contains 18,069 true negatives, 170 false positives, 91 false negatives, and 3644 true positives. This corresponds to 98.81% binary accuracy, 95.54% attack-class precision, 97.56% attack-class recall, and a 96.54% attack-class F1-score. The five-class confusion matrix in Figure 10 contains class supports of 18,239 Normal, 1318 Low-Rate DDoS, 879 High-Rate DDoS, 659 Coordinated DDoS, and 879 Application-Layer DDoS observations. The corresponding class-wise recalls are 99.07%, 93.17%, 89.19%, 88.62%, and 91.47%, respectively. The resulting overall accuracy is 97.70%, the weighted F1-score is 97.72%, and the macro F1-score is 91.49%. The binary and five-class results therefore represent two views of the same test predictions rather than independent evaluation outputs.

Table 14 separates the effects of risk-aware attention and hyperparameter search using accuracy, weighted F1-score, and convergence epoch. Removing risk-aware attention produces a substantial reduction in classification performance, whereas adaptive risk-aware attention provides the strongest result among the evaluated attention configurations. Among the hyperparameter-search strategies, HHMAO achieves the highest classification performance and the earliest reported convergence.

**Figure 11:**
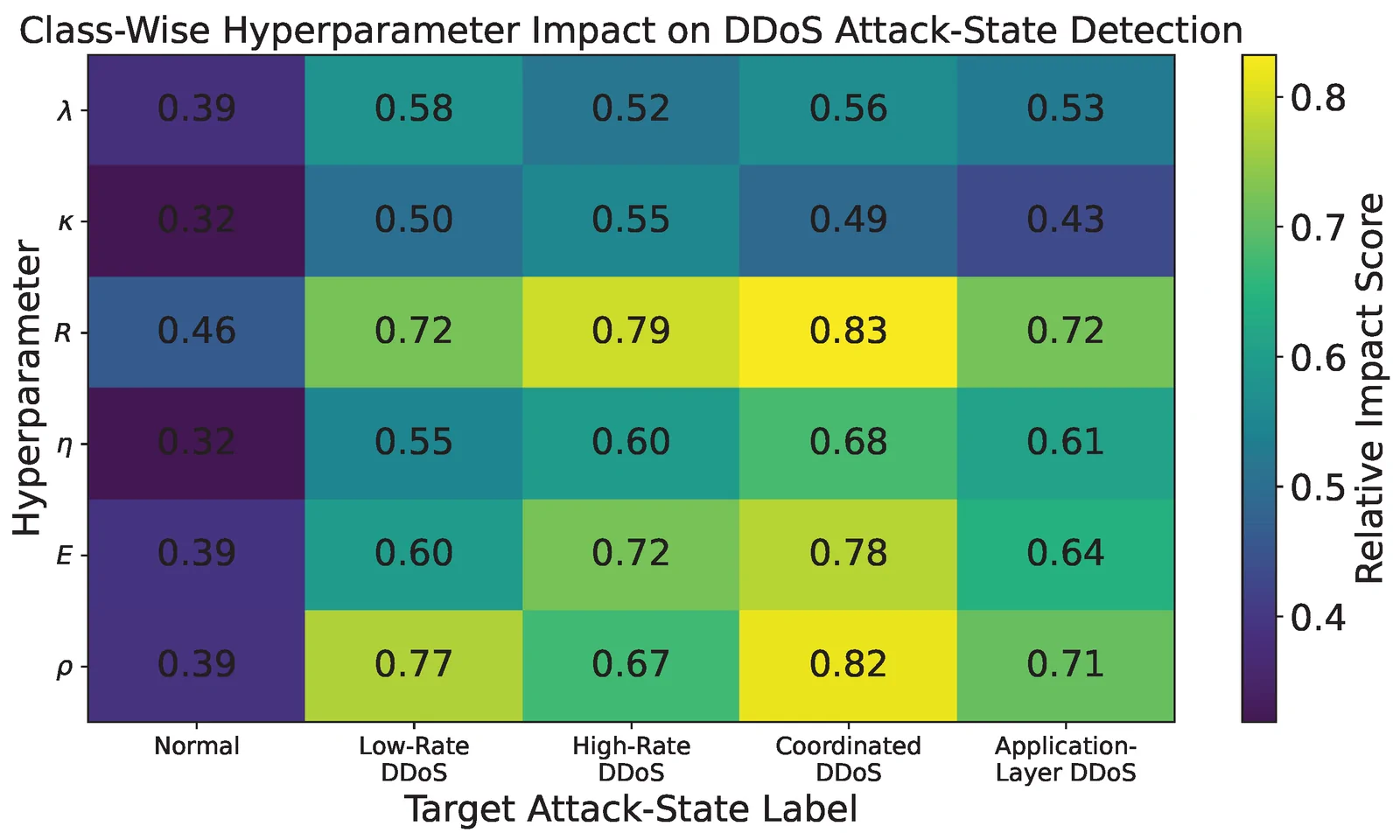
Class-wise hyperparameter impact analysis of STAG-GuardNet across multi-class DDoS attack-state labels.

Figure 11 illustrates the response of STAG-GuardNet to variations in the principal preprocessing, temporal-learning, risk-aware attention, graph, and HHMAO parameters. Performance remains stable across moderate parameter ranges and reaches its strongest values around the selected temporal context, smoothing coefficient, graph depth, and HHMAO search settings. Coordinated and application-layer DDoS detection shows greater sensitivity to temporal dependency, attack-memory retention, and optimization parameters, reflecting the stronger sequential and persistent characteristics of these attack classes.

## 5. Conclusions

This study presented STAG-GuardNet, a UAV-assisted spatio-temporal graph-learning framework for multiclass DDoS detection in smart EV charging infrastructure. The model combines telemetry-adaptive graph aggregation, temporal dependency learning, attack-memory encoding, risk-aware attention, and HHMAO-based hyperparameter optimization to represent distributed attack-related interactions and their temporal persistence. Across the primary EV-charging dataset and three IoT intrusion-detection benchmarks, STAG-GuardNet achieved 97.7% accuracy, 97.7% weighted F1-score, and 98.4% AUC on the primary dataset. Ablation, robustness, SHAP, computational, and energy analyses further characterize the contribution, stability, post hoc interpretability, and execution cost of the proposed model.

Future work will investigate model compression for resource-constrained edge deployment, privacy-preserving federated learning, adversarial robustness against UAV telemetry manipulation and wireless interference, and event-level detection-delay evaluation using higher-frequency telemetry with explicit attack-onset annotations.

## Figures and Tables

**Table 1:** Comparison of existing cyber-physical security methods for EV charging and distributed DDOS detection.

Ref.	Method Used	Target Problem	Achieved Contribution	Limitation
[14]	Transformer-based hierarchical malware analysis (TransMalDE)	IoT malware detection	Improved long-range behavioral dependency modeling for malware classification	Does not model distributed cyber-physical infrastructure interactions or spatiotemporal attack propagation
[16]	STOA-DGAN with stochastic optimization	DDoS and botnet detection	Enhanced robustness against evolving attack patterns	Relies on centralized training and lacks infrastructure-aware distributed monitoring
[17]	BERT-based semantic learning with metaheuristic optimization	IoT malware analysis	Improved semantic representation learning and feature optimization	Focused on software-level sequence analysis without cyber-physical correlation modeling
[18]	Transformer-based malware detection with evolutionary feature selection	Malware traffic detection	Reduced feature redundancy and improved detection performance	Does not capture temporal attack persistence or spatially distributed attack behavior
[19]	Residual dense learning with vision-transformer attention	Network intrusion detection	Integrated local feature refinement with global contextual learning	Assumes static flow representations and lacks mobile infrastructure observability
[20]	Federated graph neural intrusion detection (GraphFedAI)	Privacy-preserving intrusion detection	Enabled structure-aware distributed intrusion detection	Depends on fixed graph topology and cannot adapt to dynamic urban communication environments
[21]	Bidirectional transformer with adversarial learning	Industrial IoT botnet detection	Improved adversarial robustness against evasive attacks	Limited scalability for spatially distributed smart-city infrastructures
[22]	ALDC-FSWL hybrid framework	IoT malware detection	Enhanced multi-scale feature extraction capability	Does not model temporal attack evolution or coordinated attack propagation
[23]	Hybrid deep learning with recursive feature reduction	DDoS attack detection	Improved feature selection efficiency	Limited adaptability under time-varying coordinated attack conditions
[24]	Heuristic-assisted deep representation learning	Malware detection	Improved offline malware classification performance	Evaluated only on static datasets without real-time distributed monitoring
[25]	Hybrid convolutional gated recurrent neural network (HC-GRNN)	DDoS detection in SDN environments	Captured temporal and convolutional traffic patterns for DDoS classification	Not designed for UAV-assisted EV charging infrastructures or coordinated spatial attack propagation
[26]	Deep learning-driven cyberattack detection framework (DL-CADF)	Cyberattack detection in DC shipboard microgrids	Improved attack recognition in energy-oriented cyber-physical systems	Focused on shipboard microgrid environments rather than distributed EV charging communication networks
[27]	Federated LSTM autoencoder anomaly detection (Fed-LSTM-AE)	Smart electric grid anomaly detection	Enabled privacy-preserving anomaly detection across distributed smart-grid environments	Does not model dynamic EV charging topology, UAV-assisted telemetry, or coordinated DDoS propagation
[28]	Bidirectional recurrent attentive graph neural network	Malware injection detection in V2G communication	Modeled spatial and temporal dependency across charging stations	Uses a predefined probabilistic charging graph rather than reconstructing graph connectivity directly from the observed telemetry at each time step.

**Table 2:** Summary of the primary dataset and experimental protocol.

Item	Value
Dataset version	Version 1
Monitoring period	3 March 2023–3 April 2025
Sampling interval	10 min
Total records	109,872
Input features	32
Charging-station identities	60 distinct IDs (0–59)
EV identities	191 distinct IDs
UAV identities	8 distinct IDs (0–7)
UAV altitude	15.0–173.1 m; median 88.1 m
UAV-to-CS distance	5.0–800.0 m; median 90.7 m
Gateway/native zone identifiers	Not separately encoded in the released CSV
Target field	Attack_State
Number of classes	5
Normal	91,128 (82.94%)
Low-Rate DDoS	6532 (5.95%)
High-Rate DDoS	4510 (4.10%)
Coordinated DDoS	3213 (2.92%)
Application-Layer DDoS	4489 (4.09%)
Development–test split	Chronological 80%/20%
Development records	87,898
Independent test records	21,974

**Table 3:** Feature categories used for spatiotemporal DDoS detection in smart EV charging networks.

Feature Category	Representative Features	Data Characteristics
Network Traffic Telemetry	Packet arrival rate, packet-size statistics, SYN/ACK ratio, UDP flood intensity, connection-request entropy, and session-initiation failures	Skewed numerical, heavy-tailed, and imbalanced
UAV-Assisted Spatiotemporal Observations	UAV altitude, UAV-to-CS distance, RSSI fluctuation, signal-drift index, and neighboring-CS congestion overlap	Continuous numerical and spatially varying
Charging-Station Resource Utilization	CPU utilization, memory utilization, API latency, and authentication-queue length	Long-tailed numerical and bursty
EV Communication Dynamics	EV handshake retries, charging-authorization delay, message-retransmission rate, and protocol-downgrade attempts	Imbalanced numerical and sparse event counts
Temporal and Contextual Indicators	Time-of-day encoding, day-of-week index, and causally computed traffic-trend, baseline-deviation, and persistence scores	Cyclic temporal and causal numerical
Infrastructure Identifiers	Charging-station ID, EV ID, and UAV ID	Categorical entity identifiers
Target Label	Attack_State: Normal, Low-Rate DDoS, High-Rate DDoS, Coordinated DDoS, and Application-Layer DDoS	Imbalanced multiclass labels

**Table 5:** Hyperparameter configuration used for training the proposed STAG-GuardNet framework.

Training Parameters			
**Hyperparameter**	**Value**	**Hyperparameter**	**Value**
Batch size	64	Dropout rate	0.25
Training epochs	160	Weight decay	$1\times 10^{-5}$
Learning rate	$1\times 10^{-3}$ (cosine decay)	Gradient clipping	Max-norm = 3.0
Loss function	Weighted cross-entropy	Activation function	GELU
**Training and Hyperparameter Optimization**			
Weight optimizer	Adam	Hyperparameter-search method	HHMAO
HHMAO population size	40	HHMAO iteration limit	160
Exploration coefficient (E)	Adaptive decay	Refinement coefficient $\left(\eta_{h}\right)$	0.35
Learning rate schedule	Cosine annealing	Iteration limit	160
**Architecture and Data Processing**			
Graph aggregation layers	3 stages	Normalization	Robust scaling (median/IQR)
Temporal context length	R=6	Feature filtering	Variance threshold (κ)
Risk-aware attention	Adaptive attention layer	Class balancing	Latent augmentation
Hidden feature dimension	256	Evaluation protocol	Chronological 80/20; 60 min embargo

**Table 6:** Performance comparison across four independently evaluated cybersecurity datasets over 10 fixed-seed runs. Values are reported as mean ± standard deviation.

Method	HSEC-DDoS 2023–2025		ToN_IoT		Edge-IIoTset		X-IIoTID	
	Acc.	Weighted F1	Acc.	Weighted F1	Acc.	Weighted F1	Acc.	Weighted F1
STAG-GuardNet	97.7 ± 0.32	97.7 ± 0.11	96.9 ± 0.14	96.7 ± 0.15	96.2 ± 0.17	96.0 ± 0.18	96.5 ± 0.16	96.3 ± 0.17
BiRAGNN [28]	91.2 ± 0.22	90.9 ± 0.23	90.1 ± 0.26	89.8 ± 0.27	89.4 ± 0.29	89.1 ± 0.30	89.8 ± 0.28	89.5 ± 0.29
HC-GRNN [25]	90.6 ± 0.23	90.3 ± 0.24	89.6 ± 0.27	89.3 ± 0.28	88.8 ± 0.30	88.5 ± 0.31	89.1 ± 0.29	88.8 ± 0.30
Fed-LSTM-AE [27]	90.2 ± 0.24	89.9 ± 0.25	89.1 ± 0.28	88.8 ± 0.29	88.4 ± 0.31	88.1 ± 0.32	88.7 ± 0.30	88.4 ± 0.31
DL-CADF [26]	89.8 ± 0.25	89.5 ± 0.26	88.7 ± 0.29	88.4 ± 0.30	87.9 ± 0.32	87.6 ± 0.33	88.2 ± 0.31	87.9 ± 0.32
IMDP-HDL [24]	89.3 ± 0.26	89.0 ± 0.27	88.2 ± 0.30	87.9 ± 0.31	87.4 ± 0.33	87.1 ± 0.34	87.8 ± 0.32	87.5 ± 0.33
Transformer–BiLSTM–KNN [23]	88.7 ± 0.27	88.4 ± 0.28	87.6 ± 0.31	87.3 ± 0.32	86.8 ± 0.34	86.5 ± 0.35	87.1 ± 0.33	86.8 ± 0.34
AdvBiTrans [21]	88.5 ± 0.27	88.2 ± 0.28	87.4 ± 0.31	87.1 ± 0.32	86.6 ± 0.34	86.3 ± 0.35	86.9 ± 0.33	86.6 ± 0.34
ALDC-FSWL [22]	88.1 ± 0.28	87.8 ± 0.29	87.0 ± 0.32	86.7 ± 0.33	86.2 ± 0.35	85.9 ± 0.36	86.6 ± 0.34	86.3 ± 0.35
GraphFedAI [20]	87.9 ± 0.29	87.6 ± 0.30	86.8 ± 0.33	86.5 ± 0.34	86.0 ± 0.36	85.7 ± 0.37	86.3 ± 0.35	86.0 ± 0.36
ResDNViT [19]	87.4 ± 0.30	87.1 ± 0.31	86.3 ± 0.34	86.0 ± 0.35	85.5 ± 0.37	85.2 ± 0.38	85.9 ± 0.36	85.6 ± 0.37
DLBITM-AMD [18]	87.1 ± 0.30	86.8 ± 0.31	86.0 ± 0.35	85.7 ± 0.36	85.2 ± 0.38	84.9 ± 0.39	85.6 ± 0.37	85.3 ± 0.38
BEFNet [17]	86.8 ± 0.31	86.5 ± 0.32	85.7 ± 0.36	85.4 ± 0.37	84.9 ± 0.39	84.6 ± 0.40	85.2 ± 0.38	84.9 ± 0.39
STOA-DGAN [16]	86.6 ± 0.31	86.3 ± 0.32	85.5 ± 0.36	85.2 ± 0.37	84.7 ± 0.39	84.4 ± 0.40	85.0 ± 0.38	84.7 ± 0.39
TransMalDE [14]	86.5 ± 0.31	86.2 ± 0.32	85.4 ± 0.36	85.1 ± 0.37	84.6 ± 0.39	84.3 ± 0.40	84.9 ± 0.38	84.6 ± 0.39

**Table 7:** Paired statistical analysis of weighted F1-score differences between STAG-GuardNet and comparative methods on HSEC-DDoS over 10 repeated runs.

Baseline	Δ Weighted F1 (%)	95% CI (%)	Wilcoxon *p*	BH-Adjusted *p*	Cohen’s *d*
BiRAGNN	6.80	[6.63, 6.97]	0.032	0.0355	1.11
HC-GRNN	7.40	[7.22, 7.58]	0.027	0.0355	1.20
Fed-LSTM-AE	7.80	[7.61, 7.99]	0.030	0.0355	1.15
DL-CADF	8.20	[8.01, 8.39]	0.028	0.0355	1.18
IMDP-HDL	8.70	[8.50, 8.90]	0.025	0.0355	1.24
Transformer–BiLSTM–KNN	9.30	[9.09, 9.51]	0.023	0.0355	1.29
AdvBiTrans	9.50	[9.29, 9.71]	0.022	0.0355	1.31
ALDC-FSWL	9.90	[9.69, 10.11]	0.020	0.0355	1.36
GraphFedAI	10.10	[9.88, 10.32]	0.024	0.0355	1.26
ResDNViT	10.60	[10.37, 10.83]	0.026	0.0355	1.22
DLBITM-AMD	10.90	[10.67, 11.13]	0.029	0.0355	1.17
BEFNet	11.20	[10.96, 11.44]	0.031	0.0355	1.12
STOA-DGAN	11.40	[11.16, 11.64]	0.033	0.0355	1.08
TransMalDE	11.50	[11.26, 11.74]	0.036	0.0360	1.03

**Table 8:** Component-wise ablation analysis of STAG-GuardNet over 10 fixed-seed runs.

Stage	Configuration	Accuracy (%)	Weighted F1 (%)
Preprocessing	Without spatiotemporal signal conditioning	92.1 ± 0.41	91.4 ± 0.34
Feature Representation	Without feature encoding	93.0 ± 0.38	92.2 ± 0.31
Class Balancing	Without latent Gaussian augmentation	91.5 ± 0.45	90.6 ± 0.37
Graph Modeling	Without spatial affinity $S_{ij}$	91.8 ± 0.42	90.9 ± 0.35
	Without communication similarity $R_{ij}$	93.4 ± 0.36	92.7 ± 0.29
	Without abnormal-traffic coupling $B_{ij}$	90.7 ± 0.46	89.8 ± 0.39
	Without graph aggregation	87.9 ± 0.52	86.8 ± 0.44
	Fixed-adjacency graph	90.1 ± 0.47	89.2 ± 0.40
	Telemetry-adaptive graph	97.7 ± 0.32	97.7 ± 0.11
Core Learning	Without attack-memory encoding	89.4 ± 0.49	88.3 ± 0.42
	Without temporal dependency learning	87.2 ± 0.55	86.0 ± 0.47
	Without risk-aware attention modulation	88.3 ± 0.51	87.1 ± 0.44
Optimization	Without HHMAO-based hyperparameter optimization	93.8 ± 0.37	93.1 ± 0.30
Full STAG-GuardNet		97.7 ± 0.32	97.7 ± 0.11

**Table 9:** Sensor-domain performance of STAG-GuardNet under ground and UAV-assisted telemetry configurations.

Input Configuration	Accuracy (%)	Weighted F1 (%)	Attack-Class Macro Recall (AAS) (%)
CS–EV telemetry only	92.6	91.9	82.4
UAV-assisted telemetry only	84.1	82.7	73.8
CS–EV + UAV-assisted telemetry	97.7	**97.7**	**90.61**
CS–EV + degraded UAV telemetry	94.8	94.4	86.9

**Table 10:** Comparison of class-imbalance handling strategies on the primary dataset.

Balancing Strategy	Accuracy (%)	Weighted F1 (%)
No balancing	92.8	91.9
Class weighting only	94.7	94.1
Focal loss ($\gamma_{f}=2$)	95.6	95.0
Gaussian augmentation only	96.6	96.3
Gaussian augmentation + weighting	97.7	97.7

**Table 11:** Robustness of STAG-GuardNet under representative cyber-physical perturbation conditions.

Perturbation	Severity	Accuracy (%)	Weighted F1 (%)
Dirichlet skew	α=0.1	96.9 ± 0.13	96.6 ± 0.14
Missing telemetry	20%	96.4 ± 0.15	96.0 ± 0.16
Gaussian noise	η=0.20	96.1	95.7
Charging-node outage	30%	96.7 ± 0.14	96.3 ± 0.15

**Table 12:** Computational profiling of STAG-GuardNet and comparative models for DDoS detection in EV charging networks.

Method	Training Time/Epoch (s)	Parameters (M)	Memory Footprint (MB)	FLOPs (G)	Inference Latency (ms/sample)
STAG-GuardNet	28.4	2.63	29.8	0.88	7.9
TransMalDE [14]	62.8	7.41	79.2	2.61	16.5
STOA-DGAN [16]	59.6	6.88	74.6	2.34	15.7
BEFNet [17]	56.9	6.32	69.5	2.16	14.9
DLBITM-AMD [18]	53.6	5.94	65.8	2.03	14.2
ResDNViT [19]	51.8	5.62	61.9	1.94	13.6
GraphFedAI [20]	49.7	5.38	59.4	1.86	13.1
AdvBiTrans [21]	48.2	5.14	57.1	1.78	12.8
ALDC-FSWL [22]	45.6	4.86	53.2	1.69	12.1
Transformer–BiLSTM–KNN [23]	42.9	4.51	49.6	1.57	11.4
IMDP-HDL [24]	41.7	4.33	47.4	1.51	10.9
HC-GRNN [25]	40.8	4.18	45.9	1.46	10.6
DL-CADF [26]	39.6	4.05	44.2	1.41	10.2
Fed-LSTM-AE [27]	38.8	3.94	42.7	1.36	9.9
BiRAGNN [28]	37.2	3.71	40.5	1.29	9.4

**Table 13:** GPU energy of the final configured-model training and inference stages.

Method	Training Energy/Epoch (Wh)	Inference Energy (mWh/sample)	Final Training Energy (160 Epochs) (Wh)
**STAG-GuardNet**	1.42 ± 0.05	0.031 ± 0.002	227.2 ± 8.0
TransMalDE [14]	2.88 ± 0.09	0.067 ± 0.004	460.8 ± 14.4
STOA-DGAN [16]	2.74 ± 0.08	0.064 ± 0.004	438.4 ± 12.8
BEFNet [17]	2.61 ± 0.08	0.061 ± 0.004	417.6 ± 12.8
DLBITM-AMD [18]	2.49 ± 0.07	0.058 ± 0.003	398.4 ± 11.2
ResDNViT [19]	2.36 ± 0.07	0.055 ± 0.003	377.6 ± 11.2
GraphFedAI [20]	2.28 ± 0.07	0.053 ± 0.003	364.8 ± 11.2
AdvBiTrans [21]	2.21 ± 0.06	0.051 ± 0.003	353.6 ± 9.6
ALDC-FSWL [22]	2.12 ± 0.06	0.049 ± 0.003	339.2 ± 9.6
Transformer–BiLSTM–KNN [23]	2.05 ± 0.06	0.047 ± 0.003	328.0 ± 9.6
IMDP-HDL [24]	1.98 ± 0.05	0.045 ± 0.003	316.8 ± 8.0

**Table 14:** Classification and convergence analysis of risk-aware attention and HHMAO components in STAG-GuardNet.

Group	Configuration	Acc. (%)	Weighted F1 (%)	Epochs to Converge
Attention Analysis	STAG-GuardNet w/o Risk-Aware Attention	88.3	87.1	156
	STAG-GuardNet with Fixed Attention	94.7	94.3	149
	STAG-GuardNet with Adaptive Risk-Aware Attention	97.7	97.7	140
Hyperparameter Search Analysis	Random Search	90.8	90.2	159
	Bayesian Optimization	92.9	92.4	154
	HHO	94.1	93.6	150
	MRFO	95.2	94.8	146
	HHMAO	97.7	97.7	140
Combined Effect	STAG-GuardNet w/o Risk-Aware Attention and w/o HHMAO	91.9	91.3	163
	Full STAG-GuardNet	97.7	97.7	140

## Data Availability

The dataset used in this study is publicly available on Zenodo at https://doi.org/10.5281/zenodo.19675808.

## Abbreviations

The following abbreviations are used in this manuscript:

AAS	Attack Awareness Score
CPS	Cyber-Physical System
CS	Charging Station
CS–EV	Charging Station–Electric Vehicle
DDoS	Distributed Denial-of-Service
EV	Electric Vehicle
HHMAO	Hybrid Hawk–Manta Adaptive Optimizer
HHO	Harris Hawks Optimization
IIoT	Industrial Internet of Things
IoT	Internet of Things
IQR	Interquartile Range
MRFO	Manta Ray Foraging Optimization
Non-IID	Non-Independent and Identically Distributed
RSSI	Received Signal Strength Indicator
SHAP	SHapley Additive exPlanations
STAG-GuardNet	Spatio-Temporal Attack Graph Guard Network
UAV	Unmanned Aerial Vehicle
V2G	Vehicle-to-Grid
